# The small GTPase RAB-35 defines a third pathway that is required for the recognition and degradation of apoptotic cells

**DOI:** 10.1371/journal.pgen.1007558

**Published:** 2018-08-23

**Authors:** Ryan Haley, Ying Wang, Zheng Zhou

**Affiliations:** Verna and Marrs McLean Department of Biochemistry and Molecular Biology, Baylor College of Medicine, Houston, TX, United States of America; NYU School of Medicine, UNITED STATES

## Abstract

In metazoans, apoptotic cells are swiftly engulfed by phagocytes and degraded inside phagosomes. Multiple small GTPases in the Rab family are known to function in phagosome maturation by regulating vesicle trafficking. We discovered *rab-35* as a new gene important for apoptotic cell clearance from a genetic screen targeting putative Rab GTPases in *Caenorhabditis elegans*. We further identified TBC-10 as a putative GTPase-activating protein (GAP), and FLCN-1 and RME-4 as two putative Guanine Nucleotide Exchange Factors (GEFs), for RAB-35. We found that RAB-35 was required for the efficient incorporation of early endosomes to phagosomes and for the timely degradation of apoptotic cell corpses. More specifically, RAB-35 promotes two essential events that initiate phagosome maturation: the switch of phagosomal membrane phosphatidylinositol species from PtdIns(4,5)P_2_ to PtdIns(3)P, and the recruitment of the small GTPase RAB-5 to phagosomal surfaces. These functions of RAB-35 were previously unknown. Remarkably, although the phagocytic receptor CED-1 regulates these same events, RAB-35 and CED-1 appear to function independently. Upstream of degradation, RAB-35 also facilitates the recognition of apoptotic cells independently of the known CED-1 and CED-5 pathways. RAB-35 localizes to extending pseudopods and is further enriched on nascent phagosomes, consistent with its dual roles in regulating apoptotic cell-recognition and phagosome maturation. Epistasis analyses indicate that *rab-35* acts in parallel to both of the canonical *ced-1/6/7* and *ced-2/5/10/12* clearance pathways. We propose that RAB-35 acts as a robustness factor, defining a novel pathway that aids these canonical pathways in both the recognition and degradation of apoptotic cells.

## Author summary

After apoptosis, cell corpses must be promptly recognized and phagocytosed by engulfing cells. These nascent phagosomes then undergo a maturation process that results in the degradation of the apoptotic cell corpse. Phagosome maturation is enabled and coordinated through Rabs, small GTPases that recruit specific sets of effectors to the phagosome. The *ced-1/6/7/dyn-1* and *ced-2/5/10/12* pathways represent the two major canonical pathways in apoptotic cell clearance. However, substantial apoptotic cell clearance persists even when both of these pathways are inactivated. We show that the small GTPase RAB-35 promotes the recognition of cell corpses necessary for engulfment, as well as the initiation of phagosome maturation after engulfment. Specifically, RAB-35 localizes to nascent phagosomes and promotes simultaneous PtdIns(4,5)P_2_ turnover and PtdIns(3)P production, a recently discovered process that we refer to as the PtdIns(4,5)P_2_-to-PtdIns(3)P switch. We also found that RAB-35 aids in the recruitment of RAB-5 and early endosomes to the nascent phagosome. Although CED-1 also performs many of these same functions, we found that *rab-35* acts in parallel to both the *ced-1/6/7/dyn-1* and *ced-2/5/10/12* pathways. Therefore, we claim that RAB-35 defines a third pathway that functions throughout apoptotic cell clearance in order to make it more robust.

## Introduction

During the development of metazoans, cells that undergo apoptosis are internalized and degraded by other cells that are referred to as engulfing cells or phagocytes [1–3]. The phagocytic removal of apoptotic cells is an evolutionarily conserved event that supports normal tissue turnover and homeostasis, facilitates wound resolution and tissue regeneration, and prevents inflammatory and auto-immune responses induced by the release of dead cell contents [3,4]. Throughout the development of *Caenorhabditis elegans* hermaphrodites, 300–500 germ cells and 131 somatic cells undergo apoptosis [5–7]. The temporal and spatial patterns of these cell death events are highly consistent between embryos [5]. Apoptotic cells exhibit a “button-like” and highly refractive morphology under the Differential Interference Contrast (DIC) microscope, and are rapidly engulfed and degraded by multiple types of neighboring cells [5–8]. Genetic screens and further characterizations of mutations that result in the “*ce*ll *d*eath abnormal” (Ced) phenotype, characterized by the accumulation of persistent cell corpses, have identified a number of genes that act in the recognition, engulfment, or degradation of cell corpses [9,10].

The Rab family of small GTPases play critical roles in membrane trafficking events, including endocytosis and exocytosis, autophagy, and phagosome maturation [11,12]. A well-known function of Rab GTPases and their effectors is to serve as docking factors that facilitate the attachment and fusion of different membrane compartments and/or vesicles [11]. Multiple mammalian and *C*. *elegans* Rab proteins play essential roles for phagosome maturation by facilitating the incorporation of intracellular organelles to phagosomes, an action that delivers digestive enzymes to the phagosomal lumen and that might also facilitate the acidification of the lumen [13,14]. *C*. *elegans* and mammalian RAB-5 are required for the recruitment and incorporation of early endosomes to phagosomes [15–17], while *C*. *elegans* and mammalian RAB-7 are critical for the incorporation of lysosomes to phagosomes [18,19]. In *C*. *elegans*, both RAB-5 and RAB-7 function downstream of a signaling pathway that promotes phagosome maturation; this pathway is initiated by the phagocytic receptor CED-1 and mediated by the large GTPase DYN-1 [8,15,18,20]. *C*. *elegans* RAB-2 and RAB-14 also make important contributions to the phagosomal degradation of cell corpses [21–23].

The signaling pathway led by CED-1 initiates phagosome maturation not only by recruiting Rab proteins to phagosomal surfaces, but also by initiating the production of PtdIns(3)P, a phosphorylated phosphatidylinositol species and an important second messenger, on phagosomal membranes [16,18]. PtdIns(3)P recruits multiple effectors to phagosomes, including membrane remodeling factors and docking factors that facilitate the recruitment and fusion of intracellular vesicles [13,24]. Consequently, phagosome maturation events are largely dependent on the presence of PtdIns(3)P and certain Rab GTPases [15,16]. Interestingly, the presence of RAB-5 and PtdIns(3)P on phagosomal surfaces displays a co-dependent relationship [15,16].

Two phosphatidylinositol 3-kinases, PIKI-1 and VPS-34, catalyze the production of PtdIns(3)P on phagosomal surfaces [16,25]. PIKI-1 and VPS-34 are functionally opposed by MTM-1, a phosphatidylinositol 3-phosphatase that dephosphorylates PtdIns(3)P and in this manner counteracts phosphatidylinositol 3-kinase activities [16]. Throughout the phagosome maturation process, PtdIns(3)P is present on phagosomal surfaces in a two-wave oscillation pattern, a pattern coordinately regulated by PIKI-1, VPS-34, and MTM-1 [16]. MTM-1 is recruited to the surface of extending pseudopods as an effector of PtdIns(4,5)P_2_, another phosphorylated phosphatidylinositol species that is enriched on the surface of growing pseudopods during engulfment [25]. The initial appearance of PtdIns(3)P on phagosomes correlates not only with the recruitment of PIKI-1 to phagosomal surfaces by the CED-1 pathway [16], but also with the simultaneous disappearance of MTM-1 from nascent phagosomes, which is implicated to be a result of the disappearance of PtdIns(4,5)P_2_ from phagosomal surfaces [25]. Whether the CED-1 signaling pathway also regulates the turnover of PtdIns(4,5)P_2_ has not yet been tested.

The phagocytic receptor CED-1 provides a link between the engulfment of apoptotic cells and the subsequent maturation of nascent phagosomes [8,18]. During engulfment, CED-1 recognizes phosphatidylserine (PS), an “eat me” signal exposed on the surface of apoptotic cells, and defines one of the two canonical parallel pathways that stimulate pseudopod extension and cell corpse internalization [26–28]. Several other key components act in this engulfment pathway alongside CED-1: CED-7, an ABC transporter homolog that exposes PS on the surface of apoptotic cells; CED-6, a cytoplasmic adaptor for CED-1; and DYN-1, an ortholog of the large GTPase dynamin that promotes “focal exocytosis” during pseudopod extension and stabilizes the cytoskeleton underneath extending pseudopods in response to CED-1 activation [8,28,29,30]. In the other canonical engulfment pathway, CED-2 regulates the activity of the CED-5/CED-12 complex, presumably through its N-terminus that contains SH2 and SH3 domains [31–35]. The CED-5/CED-12 complex, in turn, functions as a bipartite nucleotide exchange factor to activate the Rac GTPase CED-10 [31,33]. CED-10 promotes the reorganization of the actin cytoskeleton and the extension of pseudopods around cell corpses [34,35]. However, residual engulfment activity persists after inactivating both the *ced-1/-6/-7/dyn-1* and *ced-2*/*-5/-10*/*-12* pathways, suggesting that there are yet unknown pathways that play significant roles in cell-corpse engulfment [8,36]. Although other proteins–such as alpha and beta integrins–are also reported to contribute to cell corpse engulfment, their effects are mild in comparison to the CED-1 and CED-5 pathways [37,38].

In addition to the putative missing pathways, many other questions also remain regarding the molecular mechanisms that control apoptotic cell clearance. For example, although many Rab GTPases have been implicated in the regulation of clearance events, it remains unclear whether this is an exhaustive list. The *C*. *elegans* genome contains 30 genes that encode close homologs of mammalian Rab GTPases [39,40]. To determine which of these *rab* genes function in apoptotic cell clearance, we screened for Rab GTPases that participate in cell corpse clearance by analyzing the phenotype of each candidate after RNAi knockdown or gene deletion, excluding *rab-2*, *-5*, and *-7*, which were reported to act in cell corpse clearance. We discovered that inactivation of *rab-35*, which encodes a homolog of mammalian Rab35, reduces the efficiency of apoptotic cell clearance. Further characterizations revealed novel features and functions of RAB-35. Unlike RAB-5 and RAB-7, which facilitate specific maturation events, RAB-35 regulates multiple steps throughout apoptotic cell clearance. Our findings further indicate that RAB-35 represents a clearance pathway that functions in parallel to the CED-1 and CED-5 pathways, yet in many ways resembles the mechanisms and functions of the CED-1 pathway. We thus propose that RAB-35 acts as a robustness factor, defining a novel pathway that ensures the stability of apoptotic cell clearance.

## Results

### Inactivation of *rab-35* results in an increased number of persistent cell corpses

**Fig 1:**
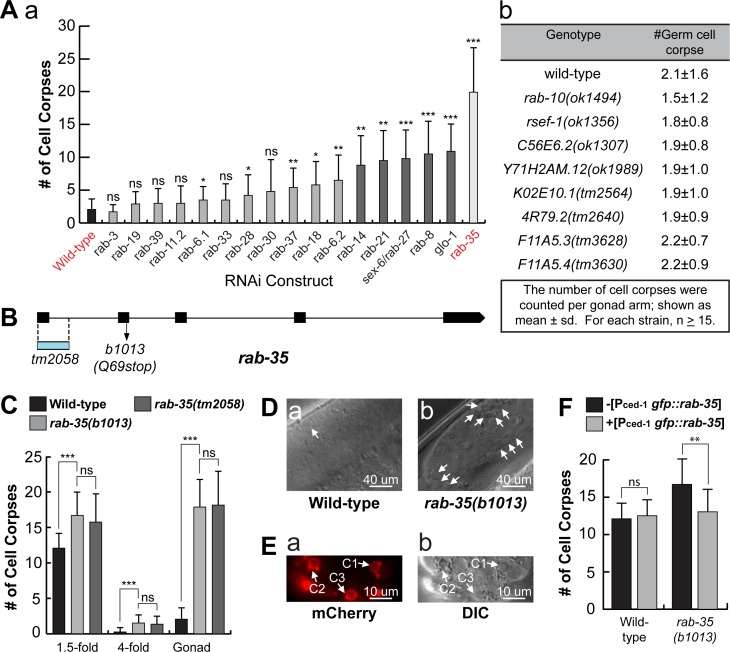
*rab-35* functions during apoptotic cell clearance in *C*. *elegans*. (A) The numbers of germ cell corpses were scored in 48-hour post-L4 adult gonads. A minimum of 15 animals were scored. Germ cell corpses were counted (a) after RNAi treatment of 17 *C*. *elegans* genes encoding RAB proteins, and (b) in wild-type and mutant alleles of *rab-10* and 7 additional candidate *rab* genes. (B) *rab-35* gene structure. Black rectangles mark exons. The blue rectangle indicates the location of the deletion in the *tm2058* allele. The arrow marks the position of the nonsense mutation carried in the *b1013* allele. (C) The numbers of cell corpses were scored at various developmental stages: 1.5-fold embryos, 4-fold embryos, and the 48-hour post-L4 adult gonad. For each data point, at least 15 animals were scored. The bars and error bars indicate mean value and standard deviation (sd), respectively. (D) Differential interference contrast (DIC) microscopy images of adult gonads. White arrows mark germ cell corpses. (E) The ventral surface of a *rab-35(b1013)* embryo that expresses MFG-E8::mcherry was visualized using both the mCherry (a) and DIC (b) channels at ~330 minutes post-first cleavage. White arrows mark the presence of MFG-E8::mcherry on C1, C2, and C3. (F) The *gfp*::*rab-35* transgene expressed in engulfing cells is able to rescue the *rab-35* mutant phenotype. The mean numbers of cell corpses in 1.5-fold stage embryos in strains carrying or not carrying P_*ced-1*_*gfp*::*rab-*35 were presented in the bar graph. For each data point, at least 15 animals were scored. Error bars represent sd. (A, C, F) Brackets above the bars indicate the samples that are compared by the Student *t*-test. p-values are summarized as such: *, 0.001 < p < 0.05; **, 0.00001 < p <0.001; ***, p <0.00001; ns, no significant difference.

The *C*. *elegans* genome contains 30 genes that encode close homologs of mammalian Rab GTPases, 23 of which have been assigned gene names [40]. Among these putative *rab* genes, *rab-2*, *rab-5*, *rab-7*, and *rab-14* have been reported to act in the clearance of apoptotic cells [13]. To determine if any other Rab proteins are involved in the same process, we individually knocked down the expression of 17 *rab* genes in *C*. *elegans* using the RNA interference (RNAi) treatment, and scored the number of germ cell corpses in the gonad of adult hermaphrodites (Materials and Methods) (Fig 1A(a)). Moreover, we scored the number of germ cell corpses in deletion mutants for additional 8 *rab* genes (Materials and Methods) (Fig 1A(b)). RNAi of *rab-1* and *rab-11*.*1* cause lethality before the worms develop into adults thus could not be scored. Of the Rabs subjected to RNAi treatment or putative loss-of-function deletions, only 6 had more than four times the number of germ cell corpses compared to the wild-type control (Fig 1A). Of these 6, *rab-35*(RNAi) worms exhibited the highest number of persistent germ cell corpses, indicating the strongest defect in the clearance of germ cell corpses, characteristic of the Ced phenotype (Fig 1A). To verify the Ced phenotype produced by *rab-35* RNAi, we examined two putative *rab-35* null alleles, the nonsense mutation *b1013* and the deletion allele *tm2058* (Fig 1B) [41]. Both *rab-35(b1013)* and *rab-35(tm2058)* mutants exhibit identical Ced phenotypes in embryos in mid- (1.5-fold, ~420 min-post 1^st^ cleavage) and late- (late 4-fold, 700–800 min-post 1^st^ cleavage) stage embryos and the 48 hour post-L4 adult gonads (Fig 1C and 1D), confirming the RNAi results.

To determine whether the button-like objects observed under DIC optics in *rab-35(b1013)* mutants are actually cell corpses, we probed them for the exposure of PS on their surfaces, a distinct characteristic of cells undergoing apoptosis [27]. Using MFG-E8::mCherry–a secreted PS-binding reporter [27], we detected bright mCherry signal specifically on the surface of the button-like objects (Fig 1E), indicating that they are indeed apoptotic cells. In addition, we expressed the *rab-35* cDNA, as an N-terminal GFP-tagged form, specifically in engulfing cells under the control of the *ced-1* promoter (P_*ced-1*_ *gfp*::*rab-35*) [26], and found that it completely rescued the Ced phenotype in *rab-35(b1013)* mutants (Fig 1F). This result suggests that RAB-35 acts in engulfing cells to promote cell corpse-clearance.

RAB-35 is known to act in receptor-mediated endocytosis and endocytic recycling in *C*. *elegans* [41,42]. In *rab-35* mutant adult hermaphrodites, we confirmed the presence of the characteristic excess of yolk in the pseudocoelom due to their previously reported defects in trafficking yolk into oocytes [41,42] (S1 Fig).

### RAB-35 localizes to developing pseudopods and must switch between its GTP- and GDP-bound forms to function

**Fig 2:**
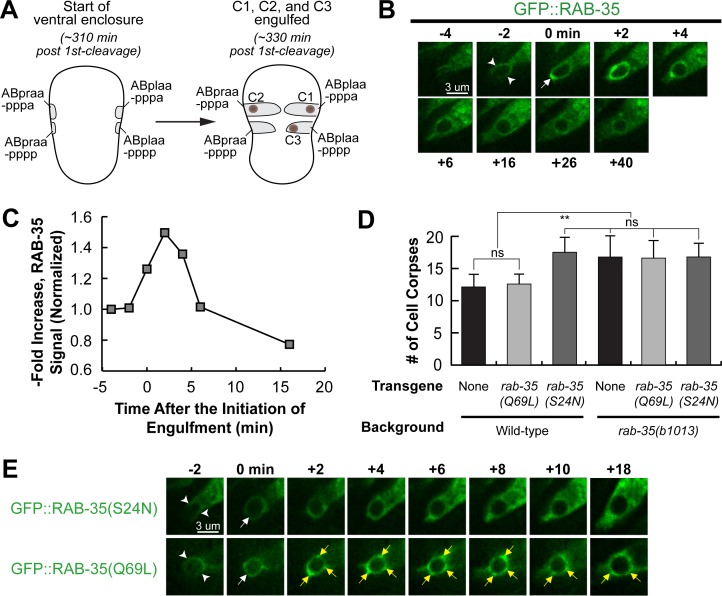
RAB-35 is localized to extending pseudopods and further enriched on nascent phagosomes. All GFP reporters are expressed in engulfing cells under the control of P_*ced-1*_. (A) Diagram illustrating the features that help us visualize ventral enclosure and apoptotic cell clearance. The start of ventral enclosure is defined as the moment the two ventral hypodermal cells (ABpraapppp and ABplaapppp) start extending to the ventral midline. Both the position of cell corpses C1, C2, and C3 (brown dots) as well as the identity of their engulfing cells are shown. (B) Time-lapse recording of GFP::RAB-35 during the engulfment and degradation of cell corpse C3 in a wild-type embryo. “0 min”: the moment a nascent phagosome is just formed. Arrowheads mark the extending pseudopods. One arrow marks the nascent phagosome. (C) Graph showing the relative GFP::RAB-35 signal intensity over time on the surface of pseudopods and the phagosome shown in B. The GFP signal intensity was measured on the phagosomal surface and in the surrounding cytoplasm every 2 minutes, starting from the “-4 min” time point. The phagosomal / cytoplasmic signal ratio over time was presented. Data is normalized relative to the signal ratio at the “-4 min” time point. (D) Bar graph presenting the mean numbers and sd (error bars) of cell corpses scored in 1.5-fold stage wild-type or *rab-35(b1013)* mutant embryos, in the presence or absence of transgenes overexpressing GFP::RAB-35(S24N) or GFP::RAB-35(Q69L). For each data point, at least 15 animals were scored. Brackets above the bars indicate the samples that are compared by the Student *t*-test. p-values are summarized as such: *, 0.001 < p < 0.05; **, 0.00001 < p <0.001; ***, p <0.00001; ns, no significant difference. (E) Time-lapse images exhibiting the localization of GFP::RAB-35(S24N) and GFP::RAB-35(Q69L) during the engulfment and degradation of C3. “0 min” is the moment a nascent phagosome is just formed. Arrowheads mark extending pseudopods. A white arrow marks the nascent phagosome. Regions with enriched GFP::RAB-35(Q69L) signal on the phagosomal membrane are marked by yellow arrows.

Using time-lapse microscopy, we monitored the localization of GFP::RAB-35 expressed in engulfing cells (P_*ced-1*_*gfp*::*rab-35*), which rescues the Ced phenotype of *rab-35(b1013)* mutants. We tracked the clearance process of three apoptotic cells on the ventral surface: C1, engulfed by ABplaapppa; C2, engulfed by ABpraapppa; and C3, engulfed by ABplaapppp, using our previously established protocol (Fig 2A) [43]. C1, C2, and C3 undergo apoptosis shortly after the initiation of ventral enclosure, an event that occurs at ~320–330 minutes post-first cleavage [43]. GFP::RAB-35 labels the extending pseudopods throughout engulfment; moreover, GFP::RAB-35 exhibits an ephemeral burst of enrichment on nascent phagosomes that lasts merely 2–4 minutes (Fig 2B). Afterwards, the phagosomal GFP signal rapidly declines to the background level by approximately 15–20 minutes after the initiation of engulfment (Fig 2B and 2C). This dynamic enrichment pattern suggests that RAB-35 might participate in multiple events during apoptotic cell clearance.

We introduced S24N and Q69L, two point mutations previously established to convert Rab GTPases into the GDP-locked and GTP-locked forms [41], respectively, individually into the P_*ced-1*_ *gfp*::*rab-35* reporter constructs. Overexpression of RAB-35(S24N) produced a Ced phenotype in the wild-type background as strong as that displayed by *rab-35* null mutants (Figs 2D and S2A), verifying its predicted dominant-negative effect [41]. Moreover, GFP::RAB-35(S24N) failed to enrich on the surfaces of extending pseudopods or nascent phagosomes (Fig 2E), suggesting that it is a non-functional form. Remarkably, overexpression of RAB-35(Q69L), the presumed GTP-locked form, failed to rescue the Ced phenotype of *rab-35* mutants (Figs 2D and S2A), although it displayed persistent enrichment on the phagosomal membrane (Fig 2E). Together, the altered localization patterns and the lack of rescuing activity observed in both mutant forms of RAB-35 suggest that the ability to switch between GTP- and GDP-bound forms is required for its proper dynamic localization pattern and function during apoptotic cell clearance.

### Determining the putative GAP and GEFs for RAB-35 for apoptotic cell clearance

**Fig 3:**
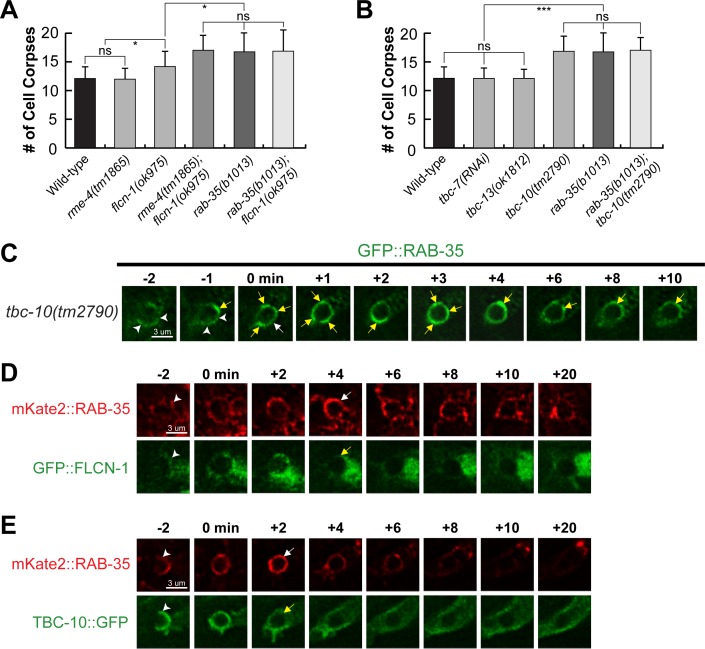
FLCN-1 and RME-4 are the candidate GEFs and TBC-10 is a candidate GAP for RAB-35. (A and B) Bar graph displaying results of the epistasis analysis between *rab-35* and genes that encode candidate GEF (A) and GAP (B) proteins for RAB-35. The mean numbers of cell corpses in 1.5-fold stage wild-type and various single and double mutant strains are presented. For each data point, at least 15 animals were scored. Error bars indicate sd. Brackets above the bars indicate the samples that are compared by the Student *t*-test. p-values are summarized as such: *, 0.001 < p < 0.05; **, 0.00001 < p <0.001; ***, p <0.00001; ns, no significant difference. (C-E): Time-lapse recording of the engulfment of C3 and the beginning of phagosome maturation in the *tbc-10(tm2790)* mutant (C) and wild-type (D-E) embryos. Reporters are indicated on the side. “0 min” is the moment a nascent phagosome is just formed. Arrowheads indicate extending pseudopods. (C) A white arrow marks the nascent phagosome. Regions on the phagosomal membrane with enriched GFP::RAB-35 signal are marked by yellow arrows. (D) White and yellow arrows each mark the burst of RAB-35 signal on and the loss of FLCN-1 signal from the phagosome, respectively. (E) White and yellow arrows each mark the burst of RAB-35 signal on and the loss of TBC-10 from the phagosome, respectively.

To better understand how the cycling of RAB-35 between the GDP- and GTP-bound forms is regulated, we examined *C*. *elegans* orthologs of known GAPs and GEFs of mammalian Rab35 to determine which ones function in the context of apoptotic cell clearance. We first studied the loss-of-function alleles of *rme-4* and *flcn-1*, which encode the *C*. *elegans* orthologs of the mammalian GEFs connecdenns 1/2/3 and folliculin, respectively [44] (S3 Fig). The *flcn-1(ok975)* null mutation resulted in a Ced phenotype that is slightly weaker than that of *rab-35(b1013)* mutants (Figs 3A and S2B). Furthermore, the *flcn-1(ok975)*; *rab-35(b1013)* double mutants exhibited a Ced phenotype identical to that of *rab-35(b1013)* mutants, placing both *flcn-1* and *rab-35* in the same genetic pathway (Figs 3A and S2B). These results suggest that *flcn-1* might act as a GEF for RAB-35, but also that it may be working in tandem with another GEF.

RME-4, a homolog of the connecdenns (S3 Fig), was previously reported to act as a GEF for RAB-35 for its function in endocytic trafficking [41]. Interestingly, although *rme-4(tm1865)* single mutants did not exhibit any statistically significant Ced phenotype, the *flcn-1(ok975)*; *rme-4(tm1865)* double mutants exhibited a Ced phenotype more severe than that displayed by the *flcn-1(ok975)* single mutants and identical in severity to that of *rab-35(b1013)* mutants (Figs 3A and S2B), suggesting that RME-4 might also function as a GEF for RAB-35 in the context of apoptotic cell clearance. Compared to FLCN-1, however, the contribution of RME-4 to apoptotic cell clearance is relatively minor and redundant.

We subsequently probed tbc-7(RNAi) and deletion mutant alleles of the genes *tbc-10*, and *tbc-13* [39], which encode the *C*. *elegans* orthologs of TBC1D24, TBC1D10A/B/C, and TBC1D13, known GAPs for mammalian Rab35, respectively [41] (S3 Fig). We found considerable evidence that TBC-10 acts as the sole GAP for RAB-35 in the context of cell corpse clearance. Firstly, loss of function of *tbc-10*, but not those of *tbc-7* or *tbc-13*, produces a Ced phenotype identical to that of *rab-35(b1013)* mutants (Figs 3B and S2C). Such results are consistent with our observation that RAB-35 must switch between its GTP- and GDP-bound forms to function (Fig 2D), as inactivating its putative GAP (*tbc-10*) appears to disable RAB-35. If the GTP-locked form of RAB-35 were its active form, *tbc-10* mutants would instead lock RAB-35 in a constitutively active form and thus fail to exhibit a Ced phenotype. Secondly, when the localization of GFP::RAB-35 throughout the clearance of C1, C2, and C3 was monitored in *tbc-10* mutants, we found that–relative to wild-type–RAB-35 was enriched on extending pseudopods and nascent phagosomes normally, but its removal from phagosomal surfaces was delayed (Fig 3C). This pattern is similar to that of GFP::RAB-35(Q69L) in a wild-type background (Fig 2E), indicating that GFP::RAB-35 is locked in the GTP-bound form in *tbc-10* mutants. Finally, the *tbc-10(tm2790); rab-35(b1013)* double mutants did not enhance the Ced phenotype over either single mutant, confirming that *tbc-10* is in the same genetic pathway as *rab-35*, as would be expected for a putative GAP for RAB-35 (Figs 3B and S2C).

To monitor the subcellular localization of FLCN-1 and TBC-10, we constructed GFP-tagged FLCN-1 and TBC-10 reporters that are expressed specifically in engulfing cells (Materials & Methods). GFP::FLCN-1 is primarily localized to the cytoplasm of the engulfing cells (Fig 3D). In addition, a weak yet visible enrichment is observed on the surface of extending pesudopods and nascent phagosomes (Fig 3D). Like GFP::FLCN-1, TBC-10::GFP also displays a transient enrichment pattern on extending pseudopods and nascent phagosomes, only the enrichment is much more distinguished (Fig 3E). Both the FLCN-1 and TBC-10 reporters colocalize with mKate2::RAB-35 during engulfment and the closure of a phagosome, yet disappear sooner from nascent phagosomes than RAB-35 (Fig 3D and 3E). These localization patterns are consistent with the model that TBC-10 and FLCN-1 act as regulators of RAB-35.

### *rab-35* loss of function causes delays in phagosomal maturation

**Fig 4:**
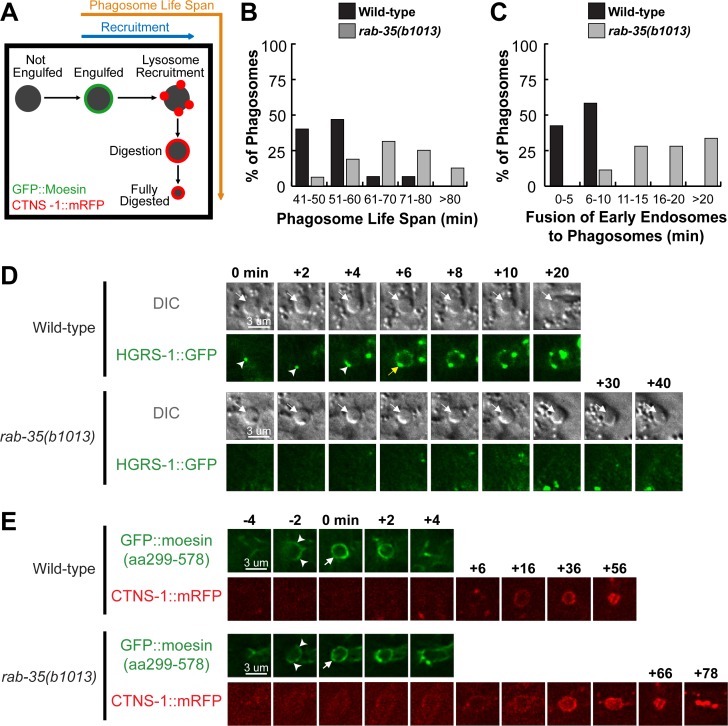
*rab-35* mutants exhibit delays in the recruitment of early endosomes, but not lysosomes, to phagosomes. (A) Diagram outlining the experiment strategy to measure the life span of a phagosome. GFP::moesin serves to mark the “0 min” time point when a phagosome is just formed, while CTNS-1::mRFP acts to track the recruitment and fusion of lysosomes to the phagosome as well as to label the phagosome during degradation. (B) Histogram displaying the life span of phagosomes bearing C1, C2, and C3 in wild-type and *rab-35(b1013)* embryos. The life span of a phagosome is defined as the time interval between the “0 min” time point and the time point when a phagosome shrinks to one-third of its radius at “0 min”. For each genotype, at least 15 phagosomes were scored. (C) Histogram displaying the range of time it takes for early endosomes to be recruited to the phagosomal surface in wild-type and *rab-35(b1013)* embryos. Phagosomes bearing C1, C2, and C3 were scored. The time interval between “0 min” and the time point when the accumulating early endosomes first form a continuous ring around a phagosome is measured and exhibited. For each genotype, at least 15 phagosomes were scored. (D) Time-lapse images monitoring the recruitment of early endosomes (reporter: HGRS-1::GFP) to the phagosomal surface after a phagosome forms (the “0 min” time point). The cell corpse (white arrows) is visualized using DIC microscopy. Arrowheads indicate HGRS-1 puncta on the phagosome. The GFP ring, when it is first completed around the phagosome, is labeled with a yellow arrow. (E) Time-lapse images showing the engulfment and degradation of a phagosome bearing C3 and the recruitment of lysosomes to phagosomal surfaces, using GFP::moesin as the pseudopod reporter and CTNS-1::mRFP as a lysosomal and phagosomal marker. “0 min” is the moment when a phagosome (white arrow) is just formed. Arrowheads indicate extending pseudopods.

*C*. *elegans* RAB-2, RAB-5, and RAB-7 all play important roles in the maturation of phagosomes that contain apoptotic cells [13]. To determine whether RAB-35 is involved in this process, we measured how fast the cell corpses C1, C2, or C3 inside phagosomes were degraded in *rab-35* mutant embryos. The lifetime of a phagosome is measured using a combination of a GFP::moesin reporter, which specifically labels the polymerized actin filaments underneath the extending pseudopods [28], and CTNS-1::mRFP, a lysosomal membrane marker that is enriched on the surface of phagosomes during maturation [18]. These two reporters are co-expressed in engulfing cells under the control of the P_*ced-1*_ promoter [18,28]. GFP::moesin is used to determine when pseudopods fuse to form a nascent phagosome, providing a way to determine the time point when phagosomal maturation begins and to measure the initial diameter of a nascent phagosome (Fig 4A). CTNS-1::mRFP is used to measure the diameter of a phagosome throughout maturation (Fig 4A). We defined phagosomal lifetime as the period it takes for the phagosome to shrink to one-third of its original radius. Using this assay, we found that *rab-35(b1013)* mutants exhibited a significantly longer phagosomal lifetime than their wild-type counterparts; 75% of phagosomes in *rab-35* mutants had a lifetime longer than 60 minutes, compared to only 13.3% of phagosomes in wild-type embryos (Fig 4B and 4E). These results indicate that RAB-35 is important for the efficient degradation of phagosomal contents.

### *rab-35* mutants are defective in the incorporation of early endosomes to phagosomes

During the degradation of cell corpses, two kinds of intracellular organelles–early endosomes and lysosomes–are known to be recruited to the surface of phagosomes and subsequently fuse to the phagosomal membrane, depositing their contents into the phagosomal lumen [13]. The recruitment and fusion of early endosomes to the phagosome was probed using HGRS-1::GFP, an established early endosomal surface marker expressed in engulfing cells [8]. Starting from the birth of a nascent phagosome, HGRS-1::GFP begins to appear on the surface of a phagosome as puncta (Fig 4D). The continuous accumulation of the puncta over time generates a GFP ring around the phagosomal surface (Fig 4D). In *rab-35* mutant embryos, this GFP ring appears much slower than in wild-type embryos; in *rab-35* mutant embryos, ~80% of the GFP rings are formed later than 10 min after a nascent phagosome forms, whereas in the wild-type background, 100% of the rings appear within 10 min. This observation suggests that RAB-35 is required for the efficient recruitment of early endosomes (Fig 4C and 4D).

CTNS-1::mRFP was used to visualize the recruitment of lysosomes to the surface of phagosomes. CTNS-1::mRFP also first appears on phagosomal surfaces as puncta, gradually accumulating and forming a mRFP ring on the phagosomal surface (Fig 4E) [18]. Time-lapse recording found that *rab-35(b1013)* mutants had no significant delays in the recruitment of lysosomes (Figs 4E and S4B). To further determine if the fusion of lysosomes to phagosomes was normal in *rab-35* mutants, we monitored the entry of a NUC-1::mRFP reporter (expressed in engulfing cells) (Materials and Methods) into the phagosomal lumen. NUC-1 is an endonuclease that specifically resides in the lysosomal lumen [45]. Similar to CTNS-1::mRFP, NUC-1::mRFP is recruited to phagosomal surfaces as mRFP^+^ puncta; however, unlike CTNS-1, the fusion of lysosomes to the phagosome causes NUC-1::mRFP to enter the phagosomal lumen (S4A Fig). In *rab-35(b1013)* embryos, both the recruitment of NUC-1::mRFP to phagosomal surfaces and entry of the mRFP signal occurred on an timescale identical to that of wild-type embryos, suggesting that inactivating *rab-35* has no effect on phagolysosomal fusion (S4B and S4C Fig).

### RAB-35 facilitates the disappearance of PtdIns(4,5)P_2_ from and production of PtdIns(3)P on phagosomal surfaces

**Fig 5:**
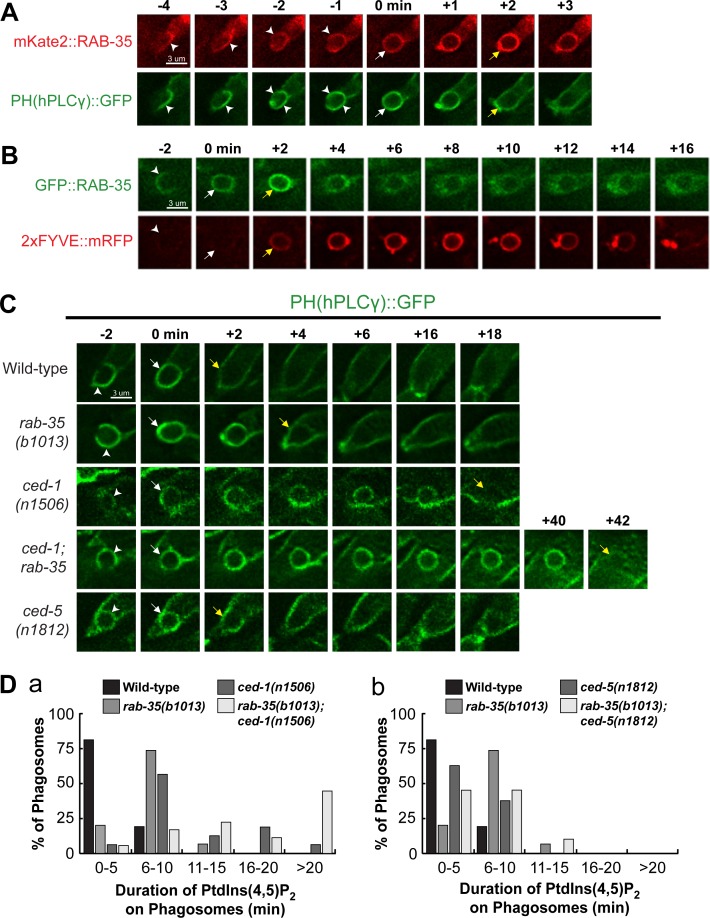
RAB-35 is enriched on phagosomal surfaces during the PtdIns(4,5)P_2_ to PtdIns(3)P shift and contributes to the PtdIns(4,5)P_2_ removal from nascent phagosomes. (A-C) Time-lapse images during and after the formation of a phagosome carrying C3 in wild-type embryos. “0 min” is the moment when a phagosome is just formed. Arrowheads indicate extending pseudopods. White arrows mark the nascent phagosome. (A) Reporters: P_*ced-1*_ *mKate2*::*rab-35* and the PtdIns(4,5)P_2_ marker P_*ced-1*_ *PH(hPLCγ)*::*gfp*. Yellow arrows mark the moment when both the gain of mKate2::RAB-35 and the loss of PH(hPLCγ)::GFP from the phagosomal surface is observed. (B) Reporters: P_*ced-1*_ *gfp*::*rab-35* and the PtdIns(3)P marker P_*ced-1*_ *2xFYVE*::*mRFP*. Yellow arrows mark the moment when both GFP::RAB-35 and 2xFYVE::mRFP are rapidly enriched on the phagosomal surface. (C) Reporter: P_*ced-1*_ *PH(hPLCγ)*::*gfp*. Yellow arrows mark the first time point when PtdIns(4,5)P_2_ is no longer observed on the phagosome surface. (D) Histograms displaying the range of time it takes for the disappearance of PtdIns(4,5)P_2_ from the surface of phagosomes bearing C1, C2, and C3 in embryos of various genotypes. The time interval between the formation of a nascent phagosome (“0 min”) and the first time point when the PH(hPLCγ)::GFP signal is no longer enriched on the phagosomal surface are displayed. For each genotype, at least 15 phagosomes were scored.

The rapid enrichment of RAB-35 during the formation of a nascent phagosome (Fig 2B) suggests that RAB-35 may function during the initiation of phagosome maturation. At this same time, the predominant phosphatidylinositol species on the phagosomal surface switches from PtdIns(4,5)P_2_ to PtdIns(3)P, a process necessary for the progression of phagosome maturation and cell corpse degradation [16,18,28]. We monitored the dynamic localization pattern of RAB-35 relative to those of PtdIns(4,5)P_2_ and PtdIns(3)P. We co-expressed, in engulfing cells, the RAB-35 reporters with previously established reporters for PtdIns(4,5)P_2_ (PH(hPLCγ)::GFP) or PtdIns(3)P (2xFYVE::mRFP) [18,28] (Fig 5A and 5B). We observed that RAB-35 enrichment corresponded exactly with both the loss of PtdIns(4,5)P_2_ and the gain of PtdIns(3)P on the surface of a nascent phagosome (Fig 5A and 5B).

Because PtdIns(3)P is essential for phagosome maturation (see Introduction), and because the disappearance of PtdIns(4,5)P_2_ from phagosomal surfaces is correlated with the production of PtdIns(3)P on phagosomes (Fig 5A and 5B) [25], we examined whether RAB-35 regulates the dynamic pattern of PtdIns(4,5)P_2_ and PtdIns(3)P on phagosomes. We first monitored PtdIns(4,5)P_2_ dynamics on the surface of phagosomes in a series of mutant embryos (Fig 5C). We found that in *rab-35(b1013)* and *ced-1(n1506)* mutants, but not *ced-5(n1812)* mutants, PtdIns(4,5)P_2_ persists longer on phagosomal surfaces (Fig 5C and 5D). *ced-1(n1506)* mutants exhibited a longer delay in PtdIns(4,5)P_2_ disappearance than *rab-35(b1013)* mutants, and *rab-35(b1013); ced-1(n1506)* double mutants exhibited a much more severe delay than either single mutant (Fig 5D(a)). These results suggest that RAB-35 and CED-1 act in a parallel and partially redundant fashion for the removal of PtdIns(4,5)P_2_ from phagosomal surfaces. The *ced-5(n1812)* null mutation, on the other hand, does not affect PtdIns(4,5)P_2_ (Fig 5C and 5D(b)).

**Fig 6:**
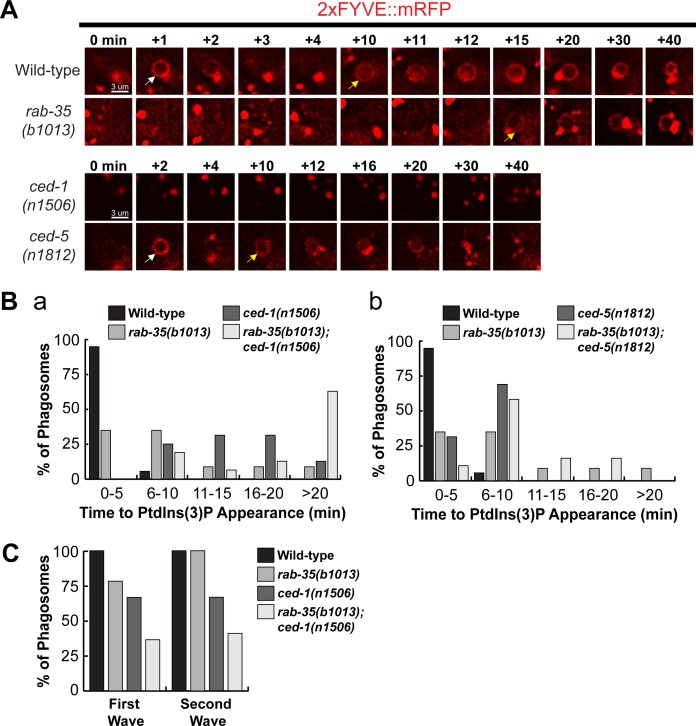
*rab-35* and *ced-1* function in parallel to produce PtdIns(3)P on the phagosomal membrane. (A) Time-lapse images during and after the formation of a phagosome carrying C3 in embryos of different genotypes expressing P_*ced-1*_*2xFYVE*::*mRFP*. “0 min” is the moment when a phagosome is just formed. White and yellow arrows indicate the time point when the 1^st^ and 2^nd^ waves of PtdIns(3)P appear on the phagosome, respectively. (B) Histogram displaying the range of time it takes for the 1^st^ peak of PtdIns(3)P to appear on phagosomes bearing C1, C2, or C3 in embryos of various genotypes, starting counting from the “0 min” time point, as shown in (A). For each genotype, at least 15 phagosomes were scored. (C) The frequency of appearance of the 1^st^ and 2^nd^ peaks of PtdIns(3)P on phagosomes carrying C1, C2, and C3 in embryos of various genotypes. For each genotype, at least 15 phagosomes were scored.

The phagocytic receptor CED-1 was previously demonstrated to play an essential role in initiating PtdIns(3)P synthesis on the surface of nascent phagosomes [18]. In *rab-35* mutant embryos, the appearance of the initial peak of PtdIns(3)P was significantly delayed compared to wild-type, although the defect was not as strong as that observed in *ced-1* mutant embryos (Fig 6A and 6B(a)). *rab-35; ced-1* double mutants have a more severe delay in the PtdIns(3)P appearance compared to either single mutant (Fig 6B(a)), again suggesting that RAB-35 and CED-1 act in parallel for the generation of PtdIns(3)P on phagosomal surfaces. On the other hand, because *ced-5* mutants fail to exhibit any delay in PtdIns(3)P production, and because the severity of the delay of PtdIns(3)P displayed by the *rab-35; ced-5* double mutants is equivalent to that of the *rab-35* single mutants, we conclude that *ced-5* is not involved in the regulation of PtdIns(3)P production (Fig 6A and 6B(b)).

PtdIns(3)P typically appears on the phagosomal surface in two distinct waves [16], as exhibited in Fig 6A (note the white and yellow arrows). In contrast to wild-type embryos, where every phagosome exhibited both waves, in *rab-35(b1013)* and *ced-1(n1506)* mutants, the first wave of PtdIns(3)P was not observed on 21.7% and 33.3% of phagosomes, respectively (Fig 6C). In addition, in *ced-1* mutants, the second wave of PtdIns(3)P was not observed 33.3% of phagosomes (Fig 6C). *rab-35(b1013)*; *ced-1(n1506)* double mutants exhibited more severe defects than either single mutant (Fig 6C). Together, our observations indicate that RAB-35 and CED-1 act in parallel to promote the production of PtdIns(3)P on the surface of nascent phagosomes.

### RAB-35 is required for the efficient removal of MTM-1 from phagosomal surfaces

**Fig 7:**
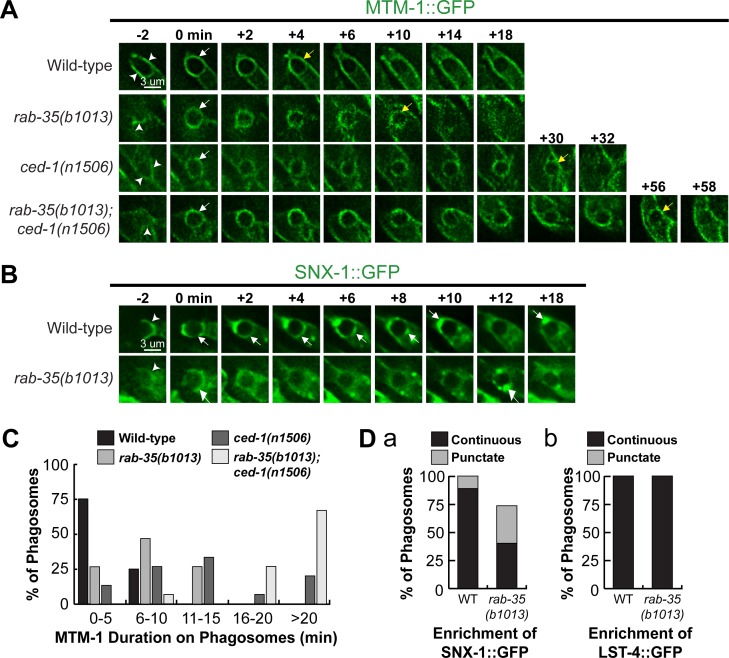
The *rab-35(b1013)* mutation impairs MTM-1 removal from, and SNX-1 recruitment to, phagosomal surfaces. (A and B) Time-lapse images during and after the formation of a phagosome carrying C3 in embryos of different genotypes expressing P_*ced-1*_ *mtm-1*::*gfp* (A) or P_*ced-1*_ *snx-1*::*gfp* (B), respectively. “0 min” is the moment when a phagosome is just formed. Arrowheads indicate extending pseudopods. White and yellow arrows on phagosomes in (A) indicate the time points when MTM-1::GFP first and last appear on the phagosome surface, respectively. White arrows in (B) mark the regions on the phagosomal surface that have an enriched GFP signal. (C) Histogram displaying the range of time that MTM-1 persists on phagosomes in wild-type and *rab-35(b1013)* embryos. Phagosomes bearing cell corpses C1, C2, and C3 were scored. This time interval is defined as that between the “0 min” time point and the first time point that MTM-1 is no longer found on the phagosome. For each genotype, at least 15 phagosomes were scored. (D) The efficiency of recruitment of SNX-1::GFP (a) and LST-4::GFP (b) to the surface of phagosomes carrying C1, C2, and C3 was scored in various genotypes. SNX-1::GFP is either distributed onto the entire phagosomal surface evenly (“continuous”) or attached to phagosomal surfaces as puncta (“punctate”) (a), whereas LST-4::GFP is enriched onto phagosomes only in the “continuous” pattern (b). For each genotype, at least 15 phagosomes were scored.

To further explore how inactivation of *rab-35* delays PtdIns(3)P production, we performed time-lapse recording of phagosomes containing C1, C2, and C3 in order to characterize the localization of PIKI-1, the PI-3 kinase that has been reported to localize to and functions on the surface of nascent phagosomes, as well as that of the PI-3 phosphatase MTM-1 that largely antagonizes PIKI-1 activity [16,25] (see Introduction). *rab-35(b1013)* mutants exhibited a normal recruitment pattern of PIKI-1::GFP; the enrichment of PIKI-1::GFP was observed on the surface of every phagosome, and the level of enrichment was comparable to that observed in wild-type embryos (S5 Fig). In contrast, MTM-1::GFP persists on the surface of phagosomes approximately twice as long in *rab-35(b1013)* as in wild-type embryos, and more than thrice as long in *ced-1(n1056)* mutants (Fig 7A). These results indicate that both RAB-35 and CED-1 are required for the timely removal of MTM-1 from phagosomal surfaces. Furthermore, *rab-35(b1013)*; *ced-1(n1506)* double mutants display an even longer delay in MTM-1::GFP removal, indicating that *rab-35* and *ced-1* function in parallel to regulate MTM-1 removal (Fig 7C). The timing of PtdIns(4,5)P_2_ disappearance and MTM-1 removal from phagosomal surfaces are similar in all backgrounds analyzed (Figs 5D and 7C), consistent with the fact that MTM-1 is a PtdIns(4,5)P_2_ effector [25]. In addition, it suggests that, in *rab-35* mutants, the persistent presence of PtdIns(4,5)P_2_ on phagosomal surfaces might cause MTM-1 to remain on phagosomes.

### RAB-35 promotes the recruitment of SNX-1, a PtdIns(3)P effector, to phagosomal surfaces

Sorting nexins SNX-1 and LST-4, two PtdIns(3)P effectors and membrane remodeling factors, are recruited to phagosomal surfaces by PtdIns(3)P [46]. SNX-1::GFP and LST-4::GFP were visualized using time lapse microscopy to characterize their localization to phagosomal surfaces. SNX-1::GFP is found on 100% of phagosomes in wild-type embryos; moreover, it forms a continuous ring on the surface of more than 90% of phagosomes, a pattern suggesting the presence of a large enough number of the SNX-1::GFP molecules to cover the entire surface of a phagosome (Fig 7B and 7D(a)). In contrast, only 73.3% of phagosomes *rab-35(b1013)* mutants recruit SNX-1::GFP; of these phagosomes, more than half recruit SNX-1::GFP as isolated puncta instead of as a continuous ring (Fig 7B and 7D(a)). These results suggest that RAB-35 is important for the efficient recruitment of SNX-1::GFP to phagosomes, consistent with the defects in PtdIns(3)P production previously observed in *rab-35* mutants. However, continuous rings of LST-4::GFP were observed on 100% of phagosomes in both *rab-35(b1013)* mutants and wild-type embryos (Fig 7D(b)). Given that the recruitment of LST-4 is mediated in part through DYN-1 [46], the mild defect in PtdIns(3)P production characteristic of *rab-35(b1013)* mutants may not be sufficient to significantly influence the recruitment of LST-4 on phagosomes.

### *rab-35* recruits RAB-5 to phagosomes and acts in the same genetic pathway as *rab-5* during phagosomal maturation

**Fig 8:**
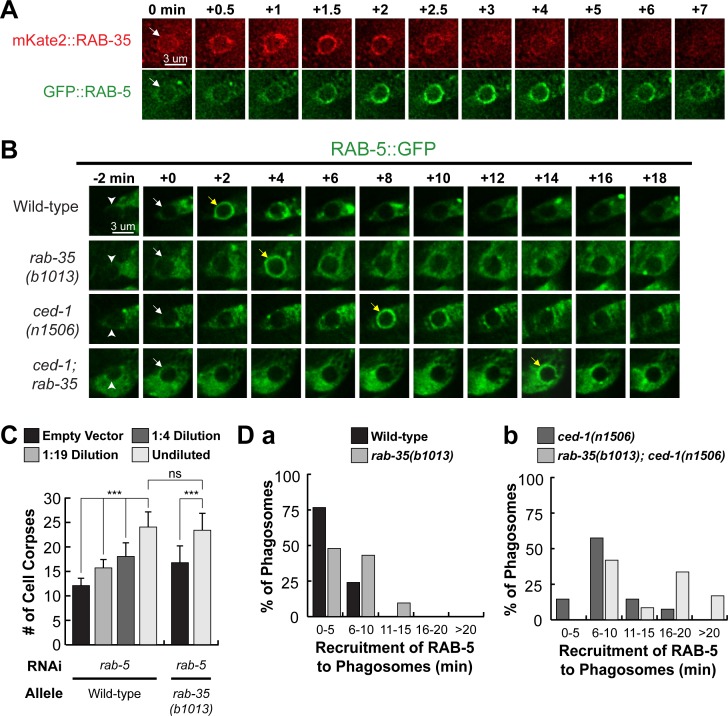
*rab-35* functions upstream of and promotes the phagosomal localization of *rab-5*. (A) Time-lapse images starting from the completion of phagosome formation (“0 min” time point) of a phagosome carrying C3 in a wild-type embryo co-expressing P_*ced-1*_ *mKate2*::*rab-35* and P_*ced-1*_ *gfp*::*rab-5*. An arrow in each strip marks the nascent phagosome. (B) Time-lapse images during and after the formation (“0 min” time point) of a phagosome carrying C3 in a wild-type embryo expressing P_*ced-1*_ *gfp*::*rab-5*. Arrowheads indicate extending pseudopods. White and yellow arrows mark the nascent phagosome and the phagosome onto which RAB-5 is first seen localized to, respectively. (C) Epistasis analysis results performed between *rab-5* and *rab-35*. *rab-5* was inactivated by feeding worms with different dilutions of *E*. *coli* carrying the RNAi construct. The numbers of cell corpses were scored in the 1.5-fold stage F1 embryos. For each data point, at least 15 embryos were scored. Mean ± sd are presented. *, 0.001 < p < 0.05; **, 0.00001 < p <0.001; ***, p <0.00001; ns, no significant difference. (D) Histograms displaying the range of time it takes for the appearance of RAB-5 on the surface of phagosomes bearing C1, C2, and C3 in embryos of various genotypes. The time interval between the “0 min” time point and the first time point that the GFP::RAB-5 signal is observed enriched on the phagosomal surface of at least 15 phagosomes for each genotype are displayed.

Given that *rab-35(b1013)* mutants are defective in production of PtdIns(3)P, and that the recruitment of RAB-5 and the production of PtdIns(3)P on phagosomal surfaces are co-dependent processes [16], we investigated the functional relationship between RAB-35 and RAB-5. We made a number of observations that indicate that RAB-35 functions upstream of RAB-5 in the regulation of phagosome maturation. Firstly, the enrichment of mKate2::RAB-35 on the surface of nascent phagosomes precedes that of GFP::RAB-5 by approximately 30–60 seconds (Fig 8A). Secondly, inactivation of *rab-5* using RNAi treatment results in the presence of extra cell corpses in an otherwise wild-type background; moreover, the *rab-35* null mutation does not further enhance the Ced phenotype caused by *rab-5*(RNAi) treatment (Figs 8C and S6), suggesting *rab-35* and *rab-5* act in the same genetic pathway. Thirdly, *rab-35(b1013)* mutants exhibit a delay in the recruitment of RAB-5 to the phagosome (Fig 8B and 8D). This delay resembles that caused by the *ced-1* mutation (Fig 8B and 8D), although it is not as severe. Additionally, *rab-35(b1013)*; *ced-1(n1506)* double mutants display a delay stronger than either single mutant (Fig 8B and 8D), suggesting that *rab-35* and *ced-1* function in parallel to recruit RAB-5 to the phagosome.

### During cell corpse internalization, RAB-35 plays a specific role in the recognition of cell corpses

**Fig 9:**
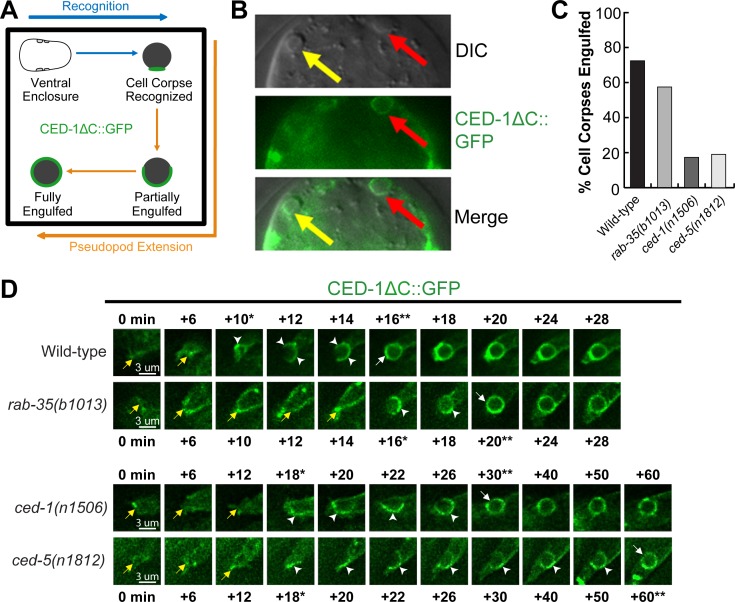
RAB-35, CED-1, and CED-5 function in parallel to recognize cell corpses. (A) Diagram outlining how the moments of cell corpse recognition and internalization are determined utilizing the CED-1ΔC::GFP reporter. The moment of recognition is defined as the first time point GFP is seen enriched in a region in contact between the engulfing and dying cell, with the moment of ventral enclosure used as a reference point (“0 min”). The time span between the moment of recognition and the moment when the nascent phagosome forms is the period of internalization. (B) Images of part of a 1.5-fold stage embryo expressing P_*ced-1*_*ced-1ΔC*::*gfp*. Cell corpses are identified under DIC optics. Red arrows mark an engulfed cell corpse, which is surrounded by a CED-1ΔC::GFP ring. Yellow arrows marks an unengulfed cell corpse, which lacks CED-1ΔC::GFP on its surface. (C) The fraction of cell corpses that have been engulfed (surrounded by a CED-1ΔC::GFP ring) in 1.5-fold to 2-fold stage embryos of various genotypes are presented as bars. For each genotype, at least 15 embryos were scored. (D) Time-lapse images before and after the formation of a phagosome carrying C3 in embryos of different genotypes expressing P_*ced-1*_*ced-1ΔC*::*gfp*. “0 min” is the moment ventral enclosure initiates. Arrowheads indicate extending pseudopods. A white arrow marks the nascent phagosome. Yellow arrows label the extending ventral hypodermal cell ABpraapppp. For each genetic background, a single asterisk marks the moment when recognition is first observed, while the double asterisks mark the moment when the nascent phagosome is formed.

The initial enrichment of GFP::RAB-35 on extending pseudopods (Fig 2B) suggests that in addition to phagosome maturation, RAB-35 might function in earlier steps of cell corpse clearance. To determine whether RAB-35 plays any role in the recognition and/or the engulfment of cell corpses, we took advantage of CED-1ΔC::GFP, a GFP tagged and truncated form of CED-1 that is missing its C-terminal intracellular domain [26]. This engulfing cell-specific transmembrane receptor first clusters to the contact site between the engulfing and dying cell, is further enriched to the extending pseudopods, and, when engulfment is complete, forms a ring around the nascent phagosome (Fig 9A) [28]. Unlike the full-length CED-1, CED-1ΔC::GFP stays on the surface of a phagosome until it is completely degraded [28]. Thus, cell corpses labeled with CED-1ΔC::GFP rings must have been previously engulfed, while cell corpses in the middle of being engulfed are labeled with partial GFP^+^ rings that represent phagocytic cups (Fig 9A). Cell corpses that are not recognized by an engulfing cell fail to be labeled by a GFP signal (Fig 9B).

We first analyzed all cell corpses in mid-stage (1.5-fold stage) embryos. In *rab-35(b1013)* embryos, a significantly lower percentage of engulfed cell corpses were observed than in wild-type embryos (Fig 9C), suggesting that the *rab-35* mutation causes defects in the internalization of cell corpses. Such a defect might result from defects in the recognition or the actual engulfment of the cell corpse. To distinguish which is the case, we monitored the recognition and engulfment of dying cells C1, C2, and C3 using the CED-1ΔC::GFP reporter. To further discern the precise moment that pseudopod extension initiates, we took advantage of the temporal consistency of *C*. *elegans* development between embryos, choosing the moment when the two ventral hypodermal cells ABplaapppp and ABpraapppp begin to extend towards the ventral midline as the “0” time point (Fig 9A). Because P_*ced-1*_ *ced-1*Δ*C*::*gfp* is expressed in embryonic hypodermal cells and localizes to the plasma membrane, the GFP signal allows us to accurately record this moment (Fig 9D). The moment of recognition is thus the time point when the clustering of GFP signal at the dying- and engulfing- cell contact site is observed.

We found that the recognition of cell corpses was delayed in *rab-35(b1013)* mutant embryos (Figs 9D and S7A). In wild-type embryos, 40% of the cell corpses are recognized within the first 10 minutes of ventral enclosure, yet in *rab-35(b1013)* mutants, only 6.7% cell corpses are recognized within the same time period (S7A(a) Fig). Additionally, in *rab-35(b1013)* mutants, 20% of the cell corpses are recognized between 21–30 minutes after the start of ventral enclosure, whereas only 6.7% of cell corpses take that long to be recognized in wild-type embryos (S7A(a) Fig). Conversely, *rab-35* null mutation causes no obvious delays in pseudopod extension and the consequential engulfment once the cell corpse is recognized (Fig 9D and S7B). Together, these results indicate that during the cell-corpse internalization process, RAB-35 specifically regulates the recognition of cell corpses.

### RAB-35 acts in a pathway separate from the CED-1 or CED-5 pathways to promote the recognition of cell corpses

We found that null mutants of *ced-1* and *ced-5*, two engulfment genes that each represents one of the two parallel pathways for engulfment [8,47,48], display significantly greater delays in cell corpse-recognition than *rab-35(b1013)* null mutants (Figs 9D and S7A(a-c)), indicating that both CED-1 and CED-5 are essential for the timely recognition of apoptotic cells. The *ced-1; ced-5* double mutant embryos suffer greater recognition delay than each single mutant strain (S7A(b-f) Fig), indicating that for the step of cell corpse-recognition, *ced-1* and *ced-5* act in two parallel pathways. We further observed that in double mutant combinations, *rab-35(b1013)* mutants enhanced the recognition delay of both *ced-1(n1506)* and *ced-5(n1812)* mutants (S7A(b-c, e) Fig), suggesting that *rab-35* acts in a pathway separate from the *ced-1* or *ced-5* pathways in the context of cell corpse recognition. The *ced-1; rab-35; ced-5* triple null mutants suffer a stronger recognition delay than any of the respective double mutants, with 63.2% of cell corpses not being recognized within 40 minutes after ventral enclosure started (S7A(d, f) Fig), further supporting our hypothesis.

The *rab-35* null mutation does not enhance the delay in engulfment caused by the *ced-1* or *ced-5* null mutations (S7B Fig), again demonstrating that *rab-35* does not regulate pseudopod extension around cell corpses.

### RAB-35 represents a novel genetic pathway that facilitates the clearance of cell corpses in parallel to the CED-1/6/7 and CED-2/5/10/12 pathways

**Fig 10:**
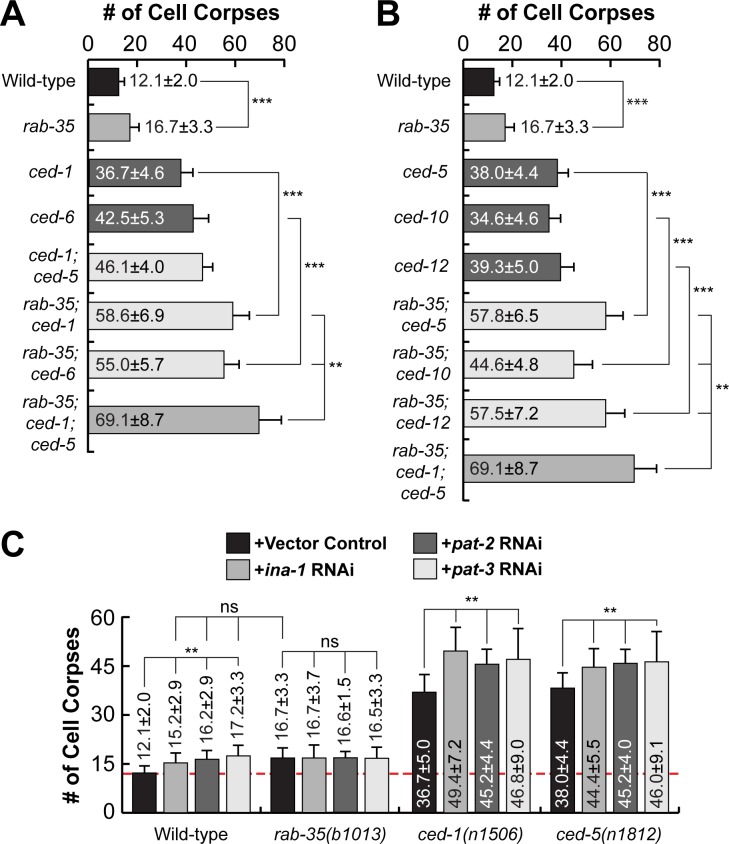
*rab-35* in an engulfment pathway independent of both the *ced-1* and *ced-5* pathways yet includes the integrins. Results of epistasis analysis performed between *rab-35* and (A) members of the *ced-1/-6/-7* pathway, (B) members of the *ced-2/-5/-10/-12* pathway, and (C) genes encoding *C*. *elegans* integrins. The average numbers of cell corpses observed in 1.5-stage embryos of various genotypes are listed and presented as bars. Error bars indicate sd. For each strain, at least 15 animals were scored. Student *t*-test was used for data analysis: *, 0.001 < p < 0.05; **, 0.00001 < p <0.001; ***, p <0.00001; ns, no significant difference. (A) Null alleles [*rab-35(b1013)*, *ced-1(n1506)*, and *ced-6(n2095)*] were used. (B) Null alleles [*ced-5(n1812)* and *ced-12(n3261)*] and a severe loss-of-function allele *ced-10*(*n1993*) were used. (C) *C*. *elegans* integrin genes *ina-1*, *pat-2*, and *cdc-42* were inactivated in wild-type, *rab-35(b1013)*, *ced-1(n1506)*, and *ced-5(n1812)* backgrounds by RNAi. A dashed line represents the mean number of cell corpses observed in untreated wild-type embryos.

We have identified the functions of RAB-35 in two distinct cell-corpse clearance events: (I) the recognition and (II) the degradation of cell corpses. In both of these events, RAB-35 appears to function independently of both CED-1 and CED-5. To further determine whether *rab-35* represents a novel pathway in addition to the *ced-1* and *ced-5* pathways to regulate apoptotic cell-clearance, we performed a thorough epistasis analysis between *rab-35* and the *ced-1*/*ced-6*/*ced-7* pathway and the *ced-2*/*ced-5*/*ced-10*/*ced-12* pathway [48] by quantifying the number of cell corpses in double mutant combinations between the putative null allele *rab-35*(*b1013)* and null alleles of representative genes in both pathways. Remarkably, *rab-35* was found to be parallel to multiple components of both the *ced-1*/*ced-6*/*ced-7* pathway (*ced-1* and *ced-6*) and the *ced-2*/*ced-5*/*ced-10*/*ced-12* pathway (*ced-5*, *ced-10*, and *ced-12*), as *rab-35* loss of function substantially enhances the Ced phenotype of each of these mutants in embryos and adult gonads (Figs 10A, 10B and S8). Furthermore, in *rab-35(b1013); ced-1(n1506)*; *ced-5(n1812)* triple mutants, the number of cell corpses is further increased relative to each of the double mutant strains (Figs 10A, 10B and S8), again suggesting that *rab-35* contributes to the clearance activity by acting in a pathway independent of either the *ced-1* or *ced-5* pathways.

To search for candidate phagocytic receptors that function in this new engulfment pathway, we examined genes encoding integrins, transmembrane receptors that have been previously implicated in apoptotic cell clearance in both mammals and *C*. *elegans* [37,38,49,50]. *C*. *elegans* contains two α integrin subunits (*ina-1*, *pat-2*) as well as a single β integrin subunit (*pat-3*), RNAi knockdown of each of which generates a modest Ced phenotype as previously reported [37,38,49,50] (Fig 10C). Furthermore, knockdown of *ina-1*, *pat-2*, or *pat-*3 fail to enhance the Ced phenotype caused by the *rab-35(b1013)* mutation (Fig 10C). On the other hand, knockdown of *ina-1*, *pat-2*, or *pat-3* all significantly enhance the Ced phenotypes caused by the *ced-1* or *ced-5* null mutations (Fig 10C). Collectively, these data indicate that integrins act in parallel to both the *ced-1* and *ced-5* pathways, but might function in the same pathway as *rab-35*.

## Discussion

Rab35 is a multifunctional GTPase that plays important roles in a wide variety of biological processes. Mammalian Rab35 has been implicated in events including, but not limited to exocytosis [51,52], endocytic recycling [53–56], cytokinesis [57,58], cytoskeleton rearrangement [58–63], and autophagy [64]. Recently, Rab35 was found to be an oncogene that promotes proliferation by activating the PI 3-kinase/AKT signaling pathway [65]. Like its mammalian homolog, *C*. *elegans* RAB-35, as well as its GEF RME-4, act in endocytic recycling and yolk uptake in developing oocytes [41].

Mammalian and *Drosophila* Rab35 have been implicated in phagocytosis and phagosome maturation, two processes that are closely related to apoptotic cell clearance. Inactivating Rab35 reduces the internalization efficiency of macrophages against erythrocytes, zymosan particles, and microbes [59,66–68]. In addition, overexpression of dominant negative Rab35(S22N) inhibits the maturation of phagosomes carrying pathogenic bacteria [69]. Although Rab35 is reported to facilitate phagocytic cup formation through ARF6 and Cdc42, both of which regulate the actin cytoskeleton [59,66,67], not much else is known about the molecular mechanisms mediated by Rab35 to regulate subsequent phagosome maturation. In addition, because these studies of mammalian Rab35 were conducted using cell cultures, the function of Rab35 during apoptotic cell clearance has yet to be explored in the context of a developing animal.

**Fig 11:**
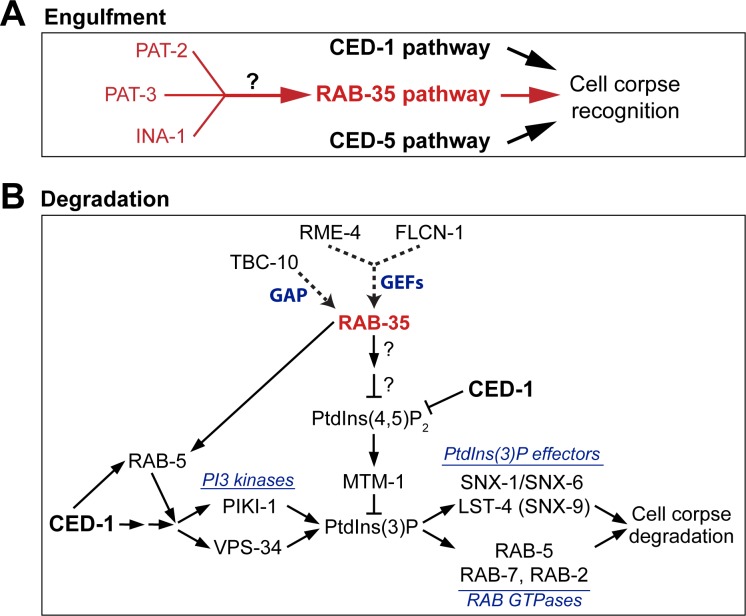
Model explaining RAB-35’s action in the clearance of apoptotic cells. Diagram illustrating the roles of RAB-35 in the engulfment (A) and degradation (B) of cell corpses in *C*. *elegans*. See Discussion for more details. RAB-35 functions in parallel with the *ced-1/-6/-7/dyn-1* and *ced-2*/*-5/-10*/*-12* pathways in the recognition of cell corpses but in the same pathway as *ina-1*, *pat-2*, and *pat-3* (A). In addition, RAB-35 functions in parallel to the CED-1 pathway during phagosome maturation (B). On the surface of nascent phagosomes, RAB-35 stimulates the turnover of both PtdIns(4,5)P_2_ and its effector MTM-1, a PI-3 phosphatase, on the phagosomal surfaces. In addition, RAB-35 helps to recruit RAB-5, which promotes the production of PtdIns(3)P. Together, these two events promote the production of phagosomal PtdIns(3)P. PtdIns(3)P in turn promotes the recruitment of its downstream effectors, driving the progression of phagosome maturation and cell corpse degradation. *rab-35* mutants are defective in the recruitment of SNX-1 but not LST-4/SNX-9 to phagosomes, perhaps due to that other factors may be involved in their recruitment. Question marks indicate that how RAB-35 regulates PtdIns(4,5)P_2_ turnover remains to be investigated.

Our work in *C*. *elegans* has discovered that RAB-35 regulates multiple apoptotic cell clearance events (Fig 11). We have uncovered a novel role of RAB-35 in the recognition of apoptotic cells, a process that enables the subsequent engulfment of apoptotic cells. RAB-35 plays an additional role in initiating the maturation of nascent phagosomes through a novel molecular mechanism that promotes the PtdIns(4,5)P_2_-to-PtdIns(3)P switch on phagosomes and facilitates the recruitment of RAB-5 to the phagosomal surface. Ultimately, RAB-35 establishes novel modes of action that ensure efficiency throughout apoptotic cell clearance.

### The function of RAB-35 depends on its cycling between GDP- and GTP-bound forms, a process facilitated by the GAP TBC-10 and the GEFs FLCN-1 and RME-4

For many Rab small GTPases, such as Rab7, the GTP- and GDP-bound forms are their active and inactive forms, respectively [18,70,71]. Other small GTPases, such as RAB-5, need to switch between the GTP- and GDP-bound forms in order to function [72]. Our characterization of the presumed GTP- and GDP-locked mutant forms of RAB-35 has revealed that the specific function of RAB-35 in apoptotic cell clearance requires it to cycle between the GTP- and GDP-bound forms, resembling the dynamics observed in RAB-5.

Among three *C*. *elegans* homologs of mammalian proteins known to act as Rab35 GAPs [73], we have identified TBC-10 as the GAP for RAB-35 in the context of apoptotic cell clearance. In *tbc-10* deletion mutants, where RAB-35 is supposedly locked in the GTP-bound state, GFP::RAB-35 persists on the surfaces of phagosomes. However, apoptotic cell clearance is defective to a degree akin to *rab-35* null mutants, supporting the hypothesis that RAB-35 must switch between its GTP- and GDP-bound forms to function properly. TBC-10 is strongly enriched on the extending pseudopods and developing phagosome and colocalizes with RAB-35. Furthermore, the disappearance of TBC-10 from phagosomal surfaces coincides in time with the burst of RAB-35 enrichment observed shortly after engulfment. TBC-10, as a GAP for RAB-35, might thus regulate multiple aspects concerning the localization and function of RAB-35, although further research is necessary.

Although *in vitro* GEF activity of folliculin for mammalian Rab35 has been detected [74], and folliculin was reported to activate Rab35 to mediate EGF receptor recycling in a cancer cell line [75], the functional relationship between folliculin and Rab35 in an animal context has not been reported. We found that *flcn-1*, the *C*. *elegans* homolog of folliculin, acts in the same genetic pathway as *rab-35* does to promote cell corpse clearance. This result, together with the observed pseudopod and phagosome enrichment of FLCN-1, suggest that FLCN-1 might act as a GEF for RAB-35 during cell corpse clearance. This is the first time that folliculin is implicated as a GEF for RAB-35 during animal development.

We have further revealed that *C*. *elegans* RME-4, a homolog of the connecdenns DENND1A-C and a GEF for RAB-35 in yolk receptor recycling [41], activates RAB-35 alongside FLCN-1 during apoptotic cell clearance. However, the *rme-4* null mutation does not cause a significant defect in clearance by itself, suggesting that FLCN-1 acts as the predominant GEF for RAB-35 in the context of apoptotic cell clearance, while RME-4 only plays a minor role. Our results are consistent with the observation that, as a multifunctional GTPase, RAB-35 is regulated by different GEFs in each cellular event that it is involved [44].

### RAB-35 modulates the initiation of phagosome maturation by regulating phosphatidylinositol dynamics and RAB-5 recruitment

Phosphorylated forms of phosphatidylinositol species are second messengers that play essential roles leading the formation and degradation of phagosomes [24]. During apoptotic cell clearance in *C*. *elegans*, a process known as PtdIns(4,5)P_2_ to PtdIns(3)P switch occurs immediately after the sealing of pseudopods and the formation of nascent phagosomes. During this switch, PtdIns(4,5)P_2_ –which has been enriched on extending pseudopods–rapidly disappears from phagosomal surfaces. PtdIns(3)P, which is essential for the initiation of phagosome maturation, subsequently appears on phagosomal surfaces at a high level and oscillates in a biphasic pattern [16,25,28].

We have observed that once engulfment starts, GFP::RAB-35 becomes enriched on the surface of extending pseudopods. The pseudopod localization pattern overlaps with that of PtdIns(4,5)P_2_ and might be a result of RAB-35’s membrane-anchoring prenylation motif typical of Rab GTPases [76] and its evolutionarily conserved polybasic region that has a high affinity for negatively charged phosphatidylinositol species such as PtdIns(4,5)P_2_ [77–79]. On the surfaces of nascent phagosomes, the further initiation of RAB-35 enrichment coincides perfectly with both the turnover of PtdIns(4,5)P_2_ and the appearance of PtdIns(3)P. This unique pattern is consistent with a role of RAB-35 in the switch of phagosomal phosphatidylinositol species from PtdIns(4,5)P_2_ to PtdIns(3)P.

Furthermore, we found that *rab-35* mutants suffer significant delays in both the disappearance of PtdIns(4,5)P_2_ and the appearance of the first wave of PtdIns(3)P on phagosomal membranes. Interestingly, we found no defects in the recruitment of the PI 3-kinase PIKI-1. However, we have discovered that the PI 3-phosphatase MTM-1, a PtdIns(4,5)P_2_ effector that dephosphorylates PtdIns(3)P and in this way counteracts the function of phosphatidylinositol 3-kinases [16,25], persists on the surface of nascent phagosomes much longer in *rab-35* mutants. Together, the above evidence indicates that RAB-35, through an unknown mechanism, promotes the turnover of PtdIns(4,5)P_2_ on phagosomal membranes, which in turn leads to the loss of MTM-1 from phagosomal membranes and the increase of PtdIns(3)P production. We have also found that RAB-35 contributes to RAB-5 recruitment on phagosomal surfaces. As RAB-5 promotes the production of phagosomal PtdIns(3)P on phagosomal surfaces [16], we propose that RAB-35 facilitates the robust production of PtdIns(3)P on phagosomal surfaces through two separate activities, the removal of PtdIns(4,5)P_2_ and the recruitment of RAB-5 (Fig 11B).

This delay of PtdIns(3)P production observed in *rab-35* mutants is associated with numerous defects in phagosome maturation: (I) the degradation of cell corpses is delayed; (II) the phagosomal recruitment of early endosomes, an intracellular organelle that is essential for phagosome maturation [13], is also delayed; and (III) SNX-1, a sorting nexin and PtdIns(3)P effector known to promote phagosome maturation [46], is recruited to the phagosomal surface less efficiently. Given that SNX-1 is necessary for the recruitment of early endosomes to phagosomes [46], we propose that this defect might be a cause for the defects in the recruitment of early endosomes. Our findings thus uncover a molecular mechanism employed by RAB-35 for cell corpse degradation and delineates a novel pathway led by RAB-35 for regulating the PtdIns(4,5)P_2_-to-PtdIns(3)P switch, an event essential for the initiation of phagosome maturation.

What we report suggests that RAB-35 promotes phagosome maturation primarily through the removal of PtdIns(4,5)P_2_ on phagosomal surfaces. Inactivation of mammalian Rab35 results in the accumulation of PtdIns(4,5)P_2_ on intracellular vacuoles and other structures [57,60], suggesting that the timely turnover of PtdIns(4,5)P_2_ is a conserved activity of Rab35. The level of PtdIns(4,5)P_2_ is known to be determined by two antagonizing activities, the phosphorylation of PtdIns(4)P or PI(5)P by PI kinases and the dephosphorylation of PtdIns(4,5)P_2_ by PI phosphatases [80]. Whether RAB-35 targets any of the PI kinases or phosphatases awaits further investigation.

### RAB-35 acts as a robustness factor and defines a novel cell corpse clearance pathway

The phagocytic receptor CED-1 and its adaptor CED-6 play a separate role in initiating phagosome maturation in addition to recognizing cell corpses and initiating engulfment [13,18]. The CED-1 pathway promotes PtdIns(3)P production on–and Rab GTPase recruitment to–phagosomal surfaces [13,18]. Here we observed that all of the defects in phagosome maturation displayed by *rab-35* null mutants, including the persistence of PtdIns(4,5)P_2_ and its effector MTM-1 on nascent phagosomes, the delay in phagosomal PtdIns(3)P production, the various defects in the recruitment of SNX-1 and RAB-5, and the delay of the incorporation of early endosomes to phagosomes, are also displayed by *ced-1* null mutants [8,18,20,46] (Figs 4–8). However, all of these defects are much more pronounced in *ced-1* mutants; for instance, phagosomal PtdIns(3)P production, the incorporation of early phagosomes, and phagosome degradation are frequently blocked in *ced-1* mutants [8,18], whereas in *rab-35* mutants they are merely delayed. In fact, this is the first time that the function of CED-1 in promoting phagosomal PtdIns(4,5)P_2_ removal is revealed.

Remarkably, *rab-35; ced-1* double mutants display enhanced defects relative to each single mutant in all of the assays mentioned above, indicating that RAB-35 initiates phagosome maturation in a manner independent of the CED-1 pathway. Given that CED-1 controls other events such as the incorporation of lysosomes to phagosomes in addition to those events regulated by RAB-35 [18], our observations suggest that this novel RAB-35 pathway acts in parallel to the CED-1 pathway in some but not necessarily all phagosome maturation events.

The recognition and engulfment of cell corpses are known to be controlled by two parallel pathways, the *ced-1/-6/-7* and *ced-2/-5/-10/-12* pathways [18,48,81]. The *rab-35(b1013)* null mutation also causes a delay in this process, but this defect is not as severe as that observed from either the *ced-1* or *ced-5* null mutations. The *rab-35* null mutation does not affect pseudopod extension, yet it enhances the defect in cell corpse recognition observed in *ced-1* or *ced-5* mutants, suggesting that RAB-35 functions in parallel to both CED-1 and CED-5 in the recognition step. When the overall clearance defects were measured by epistasis analysis, we found that *rab-35* defines a third pathway by acting in parallel to both the *ced-1/-6/-7* and *ced-2/-5/-10/-12* pathways in both the recognition and degradation of cell corpses.

INA-1, one of the two *C*. *elegans* integrin α subunits, has been proposed to act as a phagocytic receptor because of its enrichment on pseudopods and *in vivo* binding to cell corpses [37]. It was further placed in the *ced-2/-5/-10/-12* pathway through epistasis analysis [37]. PAT-2, the second integrin α subunit, and PAT-3, the β integrin subunit, were also proposed to act as phagocytic receptors, and the small GTPase CDC-42 was reported to mediate the effect of PAT-2 [38,50]. However, in the literature there is some discrepancy regarding whether PAT-2 signaling acts upstream of the CED-10/Rac1 GTPase [38,50]. Our observation that *ina-1*, *pat-2*, and *pat-3* act in the same pathway as *rab-35* and apparently in parallel to the *ced-1/-6/-7* and *ced-2/-5/-10/-12* pathways, together with the nature of integrins as transmembrane receptors, suggest that *rab-35* might function downstream of *ina-1*, *pat-2*, and *pat-3*, a hypothesis that is reflected in Fig 11. They further suggest in which pathway the integrins act might be more complex an issue than previously reported. Future investigation will determine whether and how RAB-35 mediates integrin signaling for the recognition of apoptotic cells. Also, whether the integrins participate in cell corpse-degradation needs to be explored.

Given that all phenotypes observed in *rab-35* single mutants are relatively modest, is the contribution of the *rab-35* pathway important for apoptotic cell clearance? We propose that RAB-35 acts as a robustness factor that provides a “buffer” to maintain the stability and effectiveness of cell corpse clearance. Robustness factors are important in maintaining system stability when animals encounter genetic or environmental changes. Indeed, after *rab-35* mutant embryos are subject to heat treatment, the Ced phenotype is further enhanced (S9 Fig), indicating that RAB-35 helps to keep the mechanisms behind apoptotic cell clearance stable when the system is stressed. When both the CED-1 and CED-5 pathways are intact, missing RAB-35 activity only causes modest defects, much weaker than missing either of the two canonical pathways; however, when one or both of the two canonical pathways is inactivated or when under stress, the RAB-35 pathway provides the necessary activity to support cell corpse clearance. Considering that diseases can be regarded as a subversion of the “robust yet fragile” nature of optimized and complex biological systems [82–84], we postulate that RAB-35 plays a critical role in health. This effect is likely enhanced in aging individuals that experience an increased incidence of autoimmunity and cancer [85,86], which are associated with defects in apoptotic cell clearance and RAB-35 function [3,65,73,87]. Further exploring this physiological role for RAB-35 will broaden our view of the function of Rab GTPases in both development and diseases.

## Materials and methods

### Mutations, strains, and transgenic arrays

*C*. *elegans* strains were grown at 20°C as previously described [88]. The N2 Bristol strain was used as the reference wild-type strain. Mutations and integrated arrays were performed as described previously [39,89], except when noted otherwise: LGI, *ced-1(n1506)*, *ced-12(n3261)*, *rab-10(ok1494)*, *unc-75(e950)*; LGII, *flcn-1(ok975)*, *C56E6*.*2(ok1307)*; LGIII, *ced-6(n2095)*, *rab-35(b1013*, *tm2058)*, *tbc-10(tm2790)*, *Y71H2AM*.*12(ok1989)*; LGIV, *ced-5(n1812)*, *ced-10(n1993)*, *4R79*.*2(tm2640)*; LGV, *unc-76(e911)*, *F11A5*.*3(tm3628)*, *F11A5*.*4(tm3630)*; LGX, *rme-4(ns410)*, *tbc-13(ok1812)*, *rsef-1(ok1356)*, *K02E10*.*1(tm2564)*. All *ok* alleles were provided by the *C*. *elegans* Gene Knockout Consortium and distributed by *Caenorhabditis* Genetics Center (CGC). All *tm* alleles were generated and provided by the National Bioresource Project of Japan. Transgenic lines were generated by microinjection as previously described [90]. Plasmids were injected alongside the coinjection marker pUNC76 [*unc-76(+)*] into *unc-76(e911)* mutant adult hermaphrodites as previously described [91], with non-Unc animals being identified as transgenic animals.

### Plasmid construction

The cDNAs for *rab-11*.*1*, *-18*, *-19*, *-30*. *-33*, *-35*, *glo-1*, *nuc-1*, *tbc-10*, *and flcn-1* were amplified from a mixed-stage *C*. *elegans* cDNA library using polymerase chain reaction (PCR). The cDNAs for *rab-11*.*1*, *-18*, *-19*, *-30*. *-33*, *-35*, *glo-1* were cloned into RNAi-by-feeding vector L4440 to generate RNAi constructs. P_*ced-1*_ *gfp*::*rab-35* was constructed by cloning the *rab-35* cDNAs [41] into the XmaI and KpnI sites of pZZ956 (P_*ced-1*_ *5’ gfp*). P_*ced-1*_ *mKate2*::*rab-35* was constructed by replacing the *gfp* cDNA in P_*ced-1*_ *gfp*::*rab-35* with *mKate2* cDNA [92]. The (S24N) and (Q69L) mutations were introduced into P_*ced-1*_ *gfp*::*rab-35* using the QuickChange Site-directed Mutagenesis Kit (Stratagene, La Jolla, CA) to generate P_*ced-1*_ *gfp*::*rab-35(S24N)* and P_*ced-1*_ *gfp*::*rab-35(Q69L)*, respectively. Using the same kit, the S33N mutation was introduced into P_*hsp-16/2*_ *rab-5* and P_*hsp-16/41*_ *rab-5*. P_*hsp-16/2*_ *gfp*::*rab-5(S33N)* and P_*hsp-16/41*_ *gfp*::*rab-5(S33N)* were produced by inserting the gfp cDNA into P_*hsp-16/2*_ *rab-5(S33N)* and P_*hsp-16/41*_ *rab-5(S33N)*, respectively. The *nuc-1* cDNA [93] was inserted into the BamHI and XmaI sites of pZZ829 (P_*ced-1*_ *gfp*) to generate P_*ced-1*_ *nuc-1*::*gfp*. P_*ced-1*_ *nuc-1*::*mcherry* was generated by replacing the *gfp* cDNA with the *mcherry* cDNA. P_*ced-1*_ *gfp*::*flcn-1* was constructed by cloning the *flcn-1* cDNA into the XmaI and KpnI sites of pZZ956 (P_*ced-1*_ *5’ gfp*). P_*ced-1*_ *tbc-10*::*gfp* was constructed by cloning the *tbc-10* cDNA into the AgeI and BamHI sites of pZZ829 (P_*ced-1*_ *3’ gfp*). All plasmids contain an *unc-54* 3’ UTR.

### RNA interference (RNAi)

RNAi screen of the candidate *rab* genes was performed using the feeding protocol as previously described [94]. The RNAi feeding constructs for *rab-11*.*1*, *-18*, *-19*, *-30*, *-33*, *-35*, and *glo-1* were produced by our lab, while the remaining constructs came from a *C*. *elegans* RNAi library [95,96]. Mid-L4 stage hermaphrodites were placed on plates seeded with *E*. *coli* containing the RNAi feeding construct. After 48 hrs, the numbers of germ cell corpses per gonad arm were scored using a DIC microscope.

RNAi of *tbc-7*, *rab-5*, *ina-1*, *pat-2*, *and pat-3* was performed using the same feeding protocol as above using constructs from the same library except that, 24 hrs after L4-stage hermaphrodites were placed on RNAi feeding plates, these adults were transferred to a second RNAi plate. After an additional 24 hours, the numbers of cell corpses in 1.5-fold and late 4-fold stage embryos were scored using a DIC microscope.

### Nomarski DIC microscopy

DIC microscopy was performed using an Axionplan 2 compound microscope (Carl Zeiss, Thornwood, NY) equipped with Nomarski DIC optics, a digital camera (AxioCam MRm; Carl Zeiss), and imaging software (AxioVision; Carl Zeiss). Previously established protocols were used to score cell corpses under DIC microscopy [8,43]. Somatic embryonic cell corpses were scored in the head region of embryos at various developmental stages (comma, 1.5-fold, 2-fold, late 4-fold, and early L1). Germ cell corpses were scored in one of the two gonadal arms of adult hermaphrodites 24 or 48 hrs after the mid-L4 stage. Yolk analysis was performed by characterizing the amount of yolk found in the pseudocoelom near the gonads of adult hermaphrodites 24 or 48 hrs after the L4 stage.

### Fluorescence microscopy and quantification of cell corpse clearance events

An Olympus IX70-Applied Precision DeltaVision microscope equipped with a DIC imaging apparatus and a Photometris Coolsnap 2 digital camera was used to capture fluorescence and DIC images, while Applied Precision SoftWoRx software was utilized for image deconvolution and processing [43]. To quantify the number of engulfed cell corpses in 1.5-fold to 2-fold stage embryos expressing CED-1ΔC::GFP, both DIC and GFP images of 40 serial z-sections at a 0.5-μm were recorded for each embryo. Engulfed cell corpses were those labeled with a full GFP^+^ circle. Unengulfed cell corpses were those that display the refractive appearance under DIC optics yet were either labeled with a partial GFP^+^ circle or not labeled at all.

The dynamics of various GFP, mRFP, mKate2, and mCherry reporters during the engulfment and degradation of cell corpses C1, C2, and C3 were examined using an established time-lapse recording protocol [18,43]. Ventral surfaces of embryos were initially monitored 300–320 minutes post-first cleavage. Recordings typically lasted 60–180 minutes, with an interval of 30 secs to 2 mins. At each time point, 10–16 serial z-sections at a 0.5-μm interval were recorded. Signs such as embryo elongation and embryo turning prior to comma stage were closely monitored under DIC to ensure that the embryo being recorded was developing normally. The moment of cell corpse recognition is the time when CED-1ΔC:GFP first clusters to the region where an engulfing cell contacts a cell corpse, measured relative to the moment ventral enclosure begins; the initiation of ventral enclosure is defined as the time point when hypodermal cells ABplaapppp and ABpraapppp begin to extend across the ventral surface. The time period of pseudopod extension is the time interval between when budding pseudopods labeled with CED-1ΔC::GFP are first observed and when the two pseudopods join and seal to form a nascent phagosome. The life span of a phagosome is defined as the time interval between when pseudopods seal to form the nascent phagosome and when the phagosome shrinks to one-third of its original radius.

## Supporting information

S1 Fig Loss of function of *rab-35* causes the appearance of excess yolk in the pseudocoelom. *Related to Fig 1.* (A) Differential interference contrast (DIC) microscopic images of part of adult hermaphrodites. White arrows mark pools of yolk. *rab-35(b1013)* mutants contain excess yolk in the pseudocoelom compared to wild-type. (B) A summary of the morphology and size of yolk droplets observed from wild-type and *rab-35(b1013)* mutant adults. (C) Graphs summarize the distribution of morphological classes of yolk droplets scored in 24-hour and 48-hour post-L4 wild-type and *rab-35* mutant adults. 25 individuals of each genotype were scored for each sample.
(TIF)

S2 Fig Gonadal Ced phenotype of *rab-35*(S24N) and *rab-35*(Q69L) mutant forms, *tbc-10* mutants, and *flcn-1* mutants. *Related to Figs 2 and 3.* Gonadal cell corpses were scored in one gonadal arm of each adult hermaphrodite 48 hrs-post L4 stage. Mean and sd (error bars) are presented in the bar graphs. For each sample, at least 15 animals were scored. Brackets above the bars indicate the samples that are compared by the Student *t*-test: *, 0.001 < p < 0.05; **, 0.00001 < p <0.001; ***, p <0.00001; ns, no significant difference. (A) The number of germ cell corpses in wild-type or *rab-35(b1013)* mutant adult hermaphrodites, in the presence or absence of transgenes overexpressing GFP::RAB-35(S24N) or GFP::RAB-35(Q69L). (B) The number of germ cell corpses in *flcn-1* and *rme-4* mutants and epistasis analysis between *rab-35* and these two genes. (C) The number of germ cell corpses in *tbc-10* and *tbc-13* mutants and tbc-7(RNAi) and epistasis analysis between *rab-35* and *tbc-10*.
(TIF)

S3 Fig TBC-10, RME-4, and FLCN-1 are orthologs of mammalian TBC1D10A, connecdenn 1/2/3, and folliculin, respectively. *Related to Fig 3.* All alignments were performed using EMBOSS Needle. Asterisks (*) in (A and C) and the vertical line in (B) indicate identical amino acids, colons (:) indicate similar substitutions, periods (.) indicate non-similar substitutions, and dashes (-) indicate areas where no alignment was possible. (A) Homology between TBC-10 and its human ortholog, TBC1D10A. TBC-10 and TBC1D10A share 29.6% identity and 42.3% similarity overall, and share 61.6% identity and 73.5% similarity within the highly conserved TBC (Tre-2/Bub2/Cdc16) GAP domain. The TBC domain is highlighted in yellow. The residues absent in *tbc-10(tm2790)* mutants are highlighted in red, while residues absent in *tbc-10(tm2907)* are highlighted in blue. (B) Homology between the first 500 residues of RME-4 and its human orthologs, DENND1A/connecdenn 1, DENND1B/connecdenn 2, and DENND1C/connecdenn 3 [only DENND1A is shown]. RME-4 shares 22.5% identity and 34.9% similarity overall with DENND1A; 23.6% identity and 37.4% with DENND1B; and 26.4% identity and 40.2% similarity with DENND1C. Within the more highly conserved DENN (differentially expressed in normal and neoplastic tissue) GEF domain, these values increase to 41.0% identity/67.6% similarity; 40.3% identity/66.9% similarity; and 41.7%/65.5%, respectively. The uDENN (upstream of DENN) domain is highlighted in blue, the DENN domain is highlighted in yellow, and the dDENN (downstream of DENN) domain is highlighted in green. The residues absent in *rme-4(tm1865)* mutants are highlighted in red. (C) Homology between FLCN-1 and its human ortholog folliculin. FLCN-1 and folliculin have non-canonical DENN domains, and unlike their counterparts found within RME-4 and DENND1A/B/C, they are not specifically conserved during evolution. FLCN-1 and human folliculin share 23.4% identity and 39.9% similarity overall, and 21.8% identity and 37.0% similarity with their DENN domains. The residues absent in *flcn-1(ok975)* mutants are highlighted in red.
(TIF)

S4 Fig In *rab-35(b1013)* mutants, recruitment and fusion of lysosomes to phagosomes is normal (NUC-1::mcherry). *Related to Fig 4.* (A) Time-lapse images monitoring the recruitment and fusion of lysosomes to the phagosomal surface (white arrows) after a phagosome forms (the “0 min” time point). Lysosomal fusion is monitored using NUC-1::mcherry, a lysosomal lumen marker. PH(hPLCγ)::GFP, which labels the extending pseudopods, is used to indicate the “0 min” time point when a phagosome forms. (B) Histogram displaying the range of time it takes for lysosomes to be recruited to the phagosomal surface in wild-type and *rab-35(b1013)* embryos. Phagosomes bearing cell corpses C1, C2, and C3 were scored. The time interval between “0 min” and the time point when the accumulating lysosomes first form a continuous mCherry^+^ ring around a phagosome is measured and exhibited. For each genotype, at least 15 phagosomes were scored. (C) Histogram displaying the range of time it takes for lysosomes to fuse to phagosomes in wild-type and *rab-35(b1013)* embryos. Phagosomes bearing cell corpses C1, C2, and C3 were scored. The time interval between “0 min” and the time point when the NUC-1::mCherry signal completely fills the phagosomal lumen was measured and presented. For each genotype, at least 15 phagosomes were scored.
(TIF)

S5 Fig PIKI-1 recruitment to the phagosome is normal in *rab-35(b1013)* mutants. *Related to Fig 7.* (A) Recruitment of the GFP-tagged class II PtdIns(3)P kinase GFP::PIKI-1 to nascent phagosomes was measured using live imaging of phagosomes bearing C1, C2, and C3 in wild-type and *rab-35(b1013)* mutant embryos. The presence or absence of PIKI-1 on the phagosomes was scored on each phagosome and reported as a percentage for each genetic background. There was no significant decrease in the frequency of PIKI-1 recruitment in *rab-35(b1013)* mutants. (B) During time-lapse imaging, the intensity of the PIKI-1::GFP signal was measured on the surfaces of phagosomes containing C1, C2, or C3 and in the surrounding cytoplasm at the time point of maximal PIKI-1 phagosomal signal and the ratio of signal intensity is presented in the histogram. No statistically significant changes in the relative PIKI-1 phagosomal intensity was observed in *rab-35(b1013)* mutants.
(TIF)

S6 Fig Gonadal Ced phenotype caused by RNAi knockdown of *rab-5*. *Related to Fig 8. rab-5* was inactivated by feeding wild-type or *rab-35(b1013)* mutant worms with different dilutions of *E*. *coli* carrying the *rab-5* RNAi construct. Gonadal cell corpses were scored in one gonadal arm of each adult hermaphrodite 24 hrs-post L4 stage. Mean and sd (error bars) are presented in the bar graphs. For each sample, at least 15 animals were scored. Brackets above the bars indicate the samples that are compared by the Student *t*-test: *, 0.001 < p < 0.05; **, 0.00001 < p <0.001; ***, p <0.00001; ns, no significant difference.
(TIF)

S7 Fig *rab-35(b1013)* mutants display defects in cell corpse recognition, but are normal for pseudopod extension. *Related to Fig 9.* The time it takes for engulfing cells to recognize (A) and internalize (B) cell corpses C1, C2, or C3 was determined in embryos of different genotypes using the GFP::CED-1ΔC reporter. For each strain, at least 15 engulfment events were scored. (A) The moment of recognition is defined as the first time point GFP is seen enriched in a region in contact between the engulfing and dying cell, with the moment of ventral enclosure used as a reference point (“0 min”). Histograms (a-d) and the summary (e-f) statistics are presented. (B) Internalization time is defined as the time interval between recognition of the dying cell by engulfing cells (“0 min”) and the time point that the nascent phagosome is formed. Histograms (a-d) and the summary (e-f) statistics are presented.
(TIF)

S8 Fig Gonadal Ced phenotype observed during epistasis analysis between *rab-35* and components of the *ced-1* and *ced-5* pathways. *Related to Fig 10.* Results of epistasis analysis performed between *rab-35* and the members of the *ced-1/-6/-7* (A) and the *ced-2/-5/-10/-12* (B) pathways. The mean numbers of cell corpses in the 48 hour post-L4 adult gonad of various genotypes are presented in the bar graphs. Error bars indicate sd. For each strain, at least 15 animals were scored. Student *t*-test was used for data analysis: *, 0.001 < p < 0.05; **, 0.00001 < p <0.001; ***, p <0.00001; ns, no significant difference. (A) Null alleles [*rab-35(b1013)*, *ced-1(n1506)*, and *ced-6(n2095)*] were used. (B) Null alleles [*ced-5(n1812)* and *ced-12(n3261)*] and a severe loss-of-function allele *ced-10*(*n1993*) were used.
(TIF)

S9 Fig *rab-35(b1013)* mutants display an enhanced Ced phenotype in response to heat shock treatment. The mean numbers of apoptotic cell corpses were scored in 1.5-fold stage wild-type or *rab-35(b1013)* mutant embryos carrying or not carrying a transgene overexpressing dominant negative GFP::RAB-5(S33N) under a heat shock promoter, after heat shock (33°C 2 hrs) or mock treatment. For each data point, at least 15 animals were scored. Error bars indicate sd. Student *t*-test was used for data analysis: *, 0.001 < p < 0.05; **, 0.00001 < p <0.001; ***, p <0.00001; ns, no significant difference.
(TIF)
