# Supplementary figures and images for: The small GTPase RAB-35 defines a third pathway that is required for the recognition and degradation of apoptotic cells
Source: PLoS Genet. 2018 Aug 23;14(8):e1007558. doi: 10.1371/journal.pgen.1007558 (PMC6107108; doi:10.1371/journal.pgen.1007558)

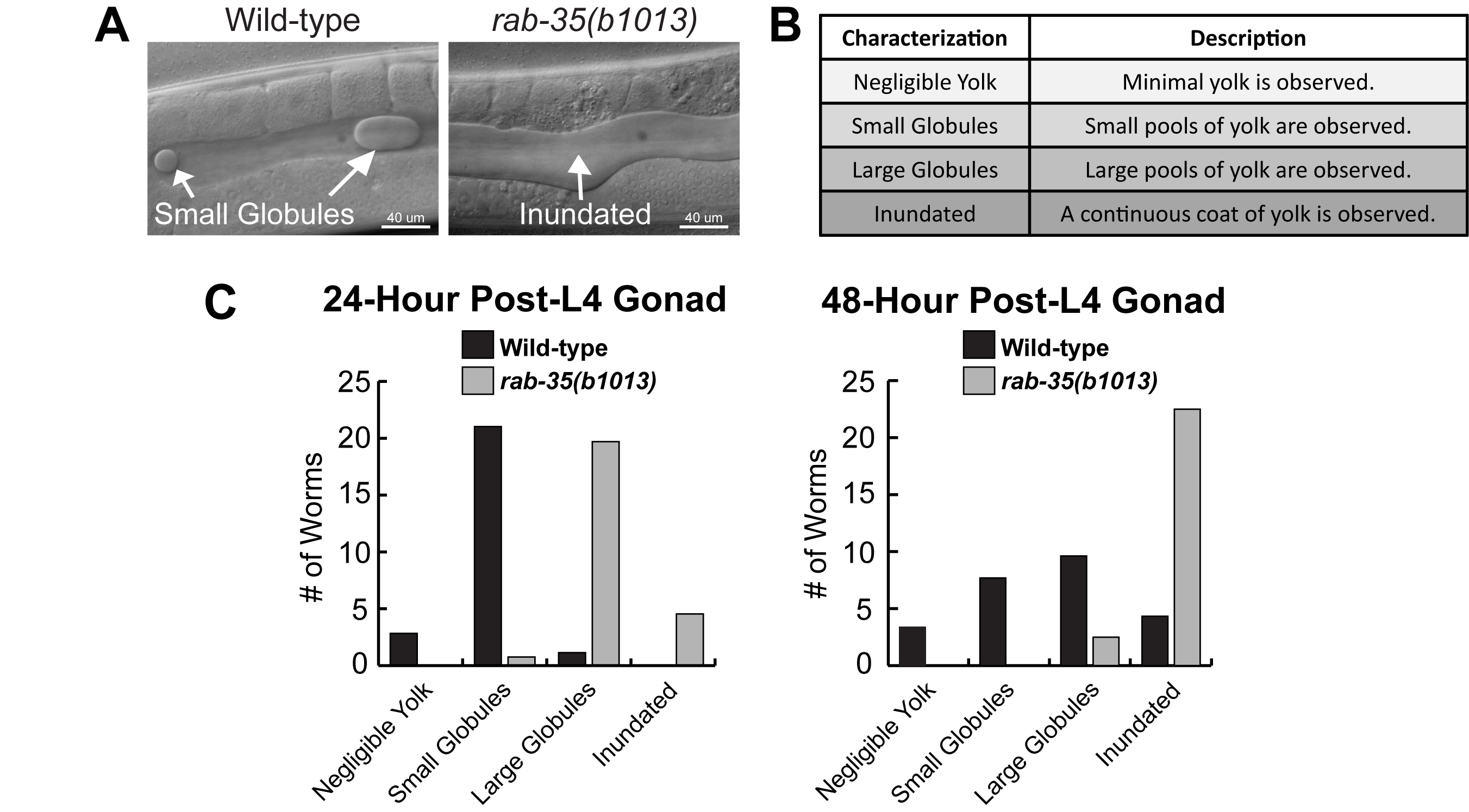

Supplement: S1 Fig — Related to Fig 1. (A) Differential interference contrast (DIC) microscopic images of part of adult hermaphrodites. White arrows mark pools of yolk. rab-35(b1013) mutants contain excess yolk in the pseudocoelom compared to wild-type. (B) A summary of the morphology and size of yolk droplets observed from wild-type and rab-35(b1013) mutant adults. (C) Graphs summarize the distribution of morphological classes of yolk droplets scored in 24-hour and 48-hour post-L4 wild-type and rab-35 mutant adults. 25 individuals of each genotype were scored for each sample. (TIF) [file pgen.1007558.s001.tif]

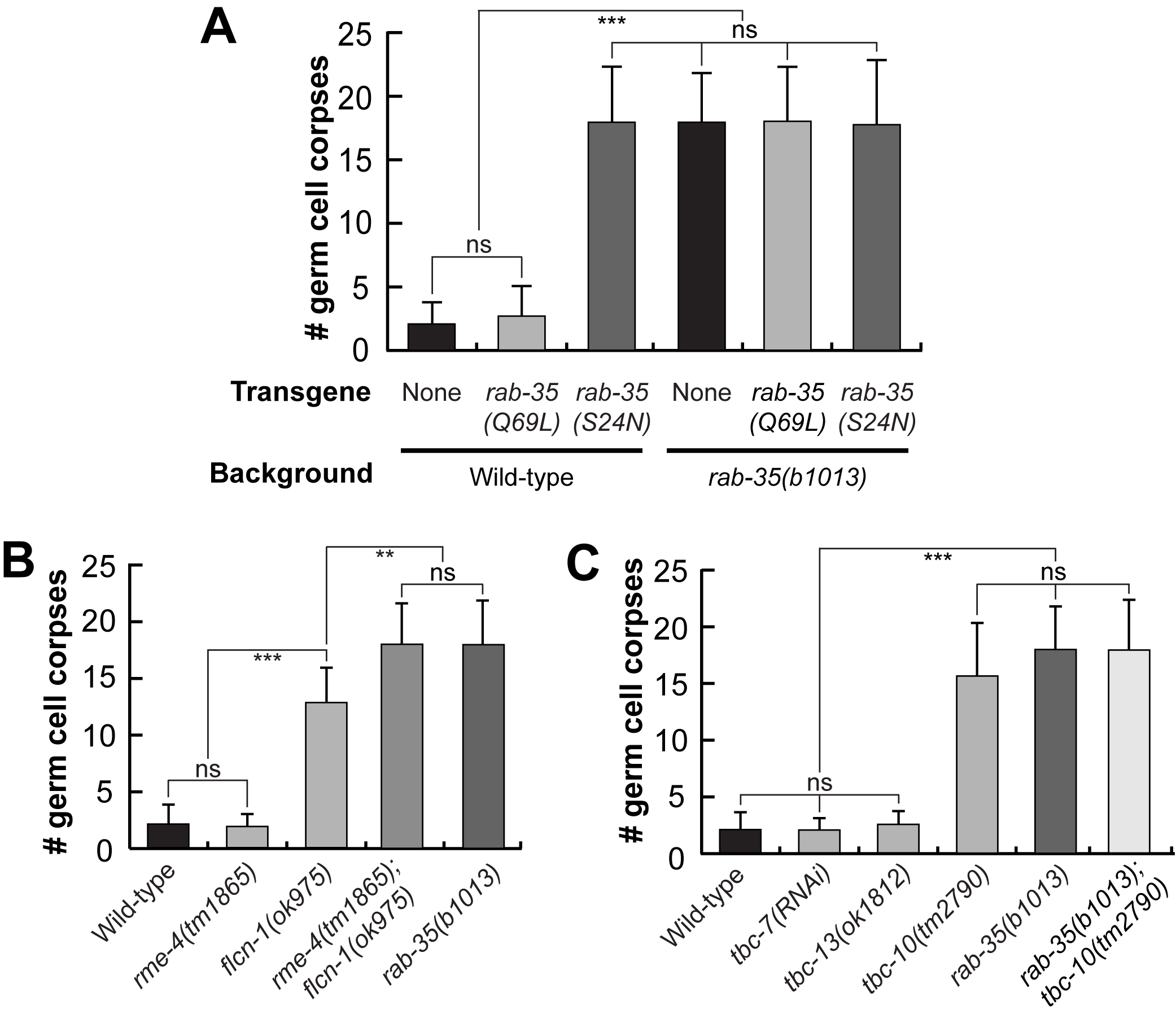

Supplement: S2 Fig — Related to Figs 2 and 3. Gonadal cell corpses were scored in one gonadal arm of each adult hermaphrodite 48 hrs-post L4 stage. Mean and sd (error bars) are presented in the bar graphs. For each sample, at least 15 animals were scored. Brackets above the bars indicate the samples that are compared by the Student t-test: *, 0.001 < p < 0.05; **, 0.00001 < p <0.001; ***, p <0.00001; ns, no significant difference. (A) The number of germ cell corpses in wild-type or rab-35(b1013) mutant adult hermaphrodites, in the presence or absence of transgenes overexpressing GFP::RAB-35(S24N) or GFP::RAB-35(Q69L). (B) The number of germ cell corpses in flcn-1 and rme-4 mutants and epistasis analysis between rab-35 and these two genes. (C) The number of germ cell corpses in tbc-10 and tbc-13 mutants and tbc-7(RNAi) and epistasis analysis between rab-35 and tbc-10. (TIF) [file pgen.1007558.s002.tif]

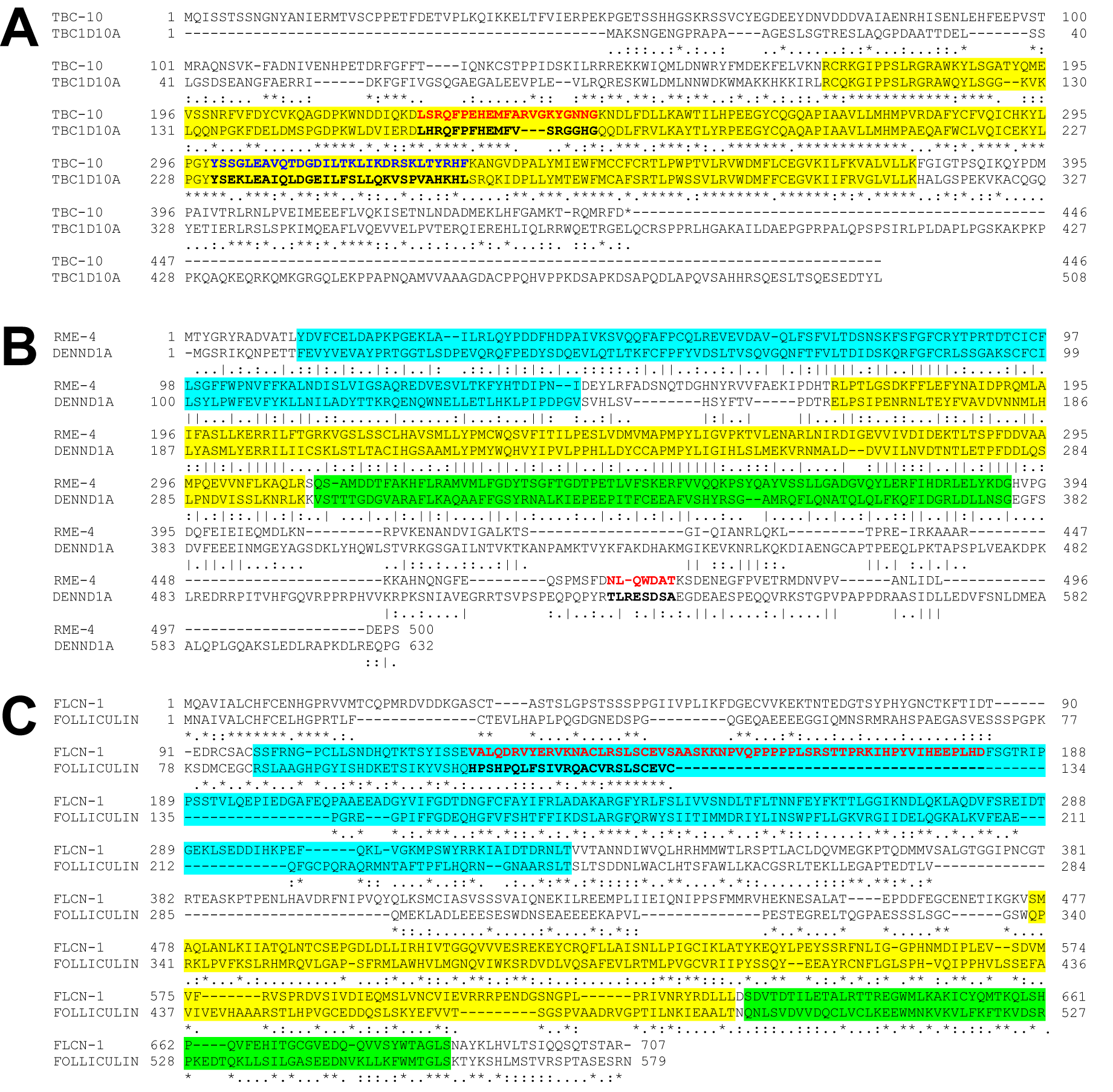

Supplement: S3 Fig — Related to Fig 3. All alignments were performed using EMBOSS Needle. Asterisks (*) in (A and C) and the vertical line in (B) indicate identical amino acids, colons (:) indicate similar substitutions, periods (.) indicate non-similar substitutions, and dashes (-) indicate areas where no alignment was possible. (A) Homology between TBC-10 and its human ortholog, TBC1D10A. TBC-10 and TBC1D10A share 29.6% identity and 42.3% similarity overall, and share 61.6% identity and 73.5% similarity within the highly conserved TBC (Tre-2/Bub2/Cdc16) GAP domain. The TBC domain is highlighted in yellow. The residues absent in tbc-10(tm2790) mutants are highlighted in red, while residues absent in tbc-10(tm2907) are highlighted in blue. (B) Homology between the first 500 residues of RME-4 and its human orthologs, DENND1A/connecdenn 1, DENND1B/connecdenn 2, and DENND1C/connecdenn 3 [only DENND1A is shown]. RME-4 shares 22.5% identity and 34.9% similarity overall with DENND1A; 23.6% identity and 37.4% with DENND1B; and 26.4% identity and 40.2% similarity with DENND1C. Within the more highly conserved DENN (differentially expressed in normal and neoplastic tissue) GEF domain, these values increase to 41.0% identity/67.6% similarity; 40.3% identity/66.9% similarity; and 41.7%/65.5%, respectively. The uDENN (upstream of DENN) domain is highlighted in blue, the DENN domain is highlighted in yellow, and the dDENN (downstream of DENN) domain is highlighted in green. The residues absent in rme-4(tm1865) mutants are highlighted in red. (C) Homology between FLCN-1 and its human ortholog folliculin. FLCN-1 and folliculin have non-canonical DENN domains, and unlike their counterparts found within RME-4 and DENND1A/B/C, they are not specifically conserved during evolution. FLCN-1 and human folliculin share 23.4% identity and 39.9% similarity overall, and 21.8% identity and 37.0% similarity with their DENN domains. The residues absent in flcn-1(ok975) mutants are highlighted in red. (TIF) [file pgen.1007558.s003.tif]

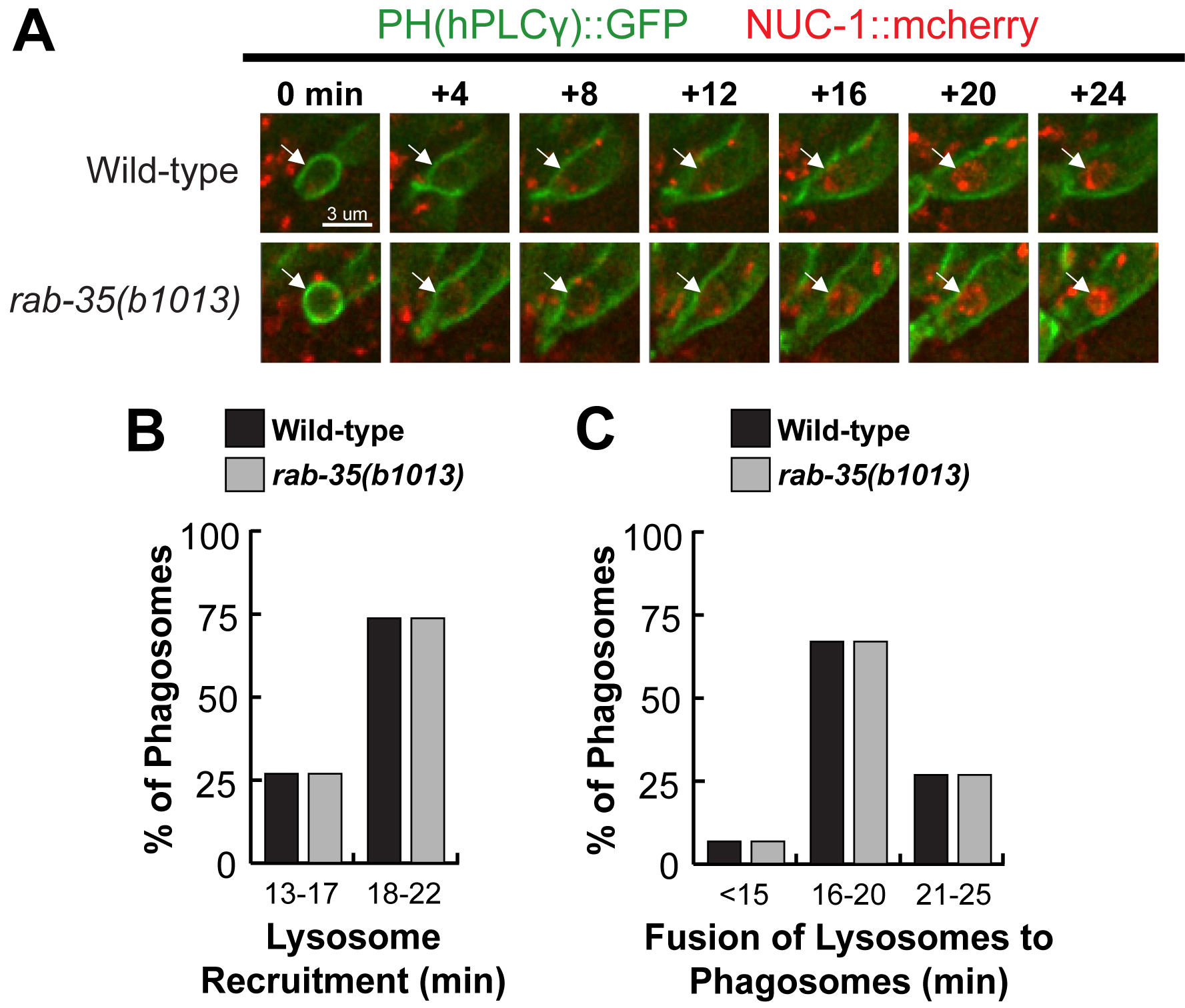

Supplement: S4 Fig — Related to Fig 4. (A) Time-lapse images monitoring the recruitment and fusion of lysosomes to the phagosomal surface (white arrows) after a phagosome forms (the “0 min” time point). Lysosomal fusion is monitored using NUC-1::mcherry, a lysosomal lumen marker. PH(hPLCγ)::GFP, which labels the extending pseudopods, is used to indicate the “0 min” time point when a phagosome forms. (B) Histogram displaying the range of time it takes for lysosomes to be recruited to the phagosomal surface in wild-type and rab-35(b1013) embryos. Phagosomes bearing cell corpses C1, C2, and C3 were scored. The time interval between “0 min” and the time point when the accumulating lysosomes first form a continuous mCherry+ ring around a phagosome is measured and exhibited. For each genotype, at least 15 phagosomes were scored. (C) Histogram displaying the range of time it takes for lysosomes to fuse to phagosomes in wild-type and rab-35(b1013) embryos. Phagosomes bearing cell corpses C1, C2, and C3 were scored. The time interval between “0 min” and the time point when the NUC-1::mCherry signal completely fills the phagosomal lumen was measured and presented. For each genotype, at least 15 phagosomes were scored. (TIF) [file pgen.1007558.s004.tif]

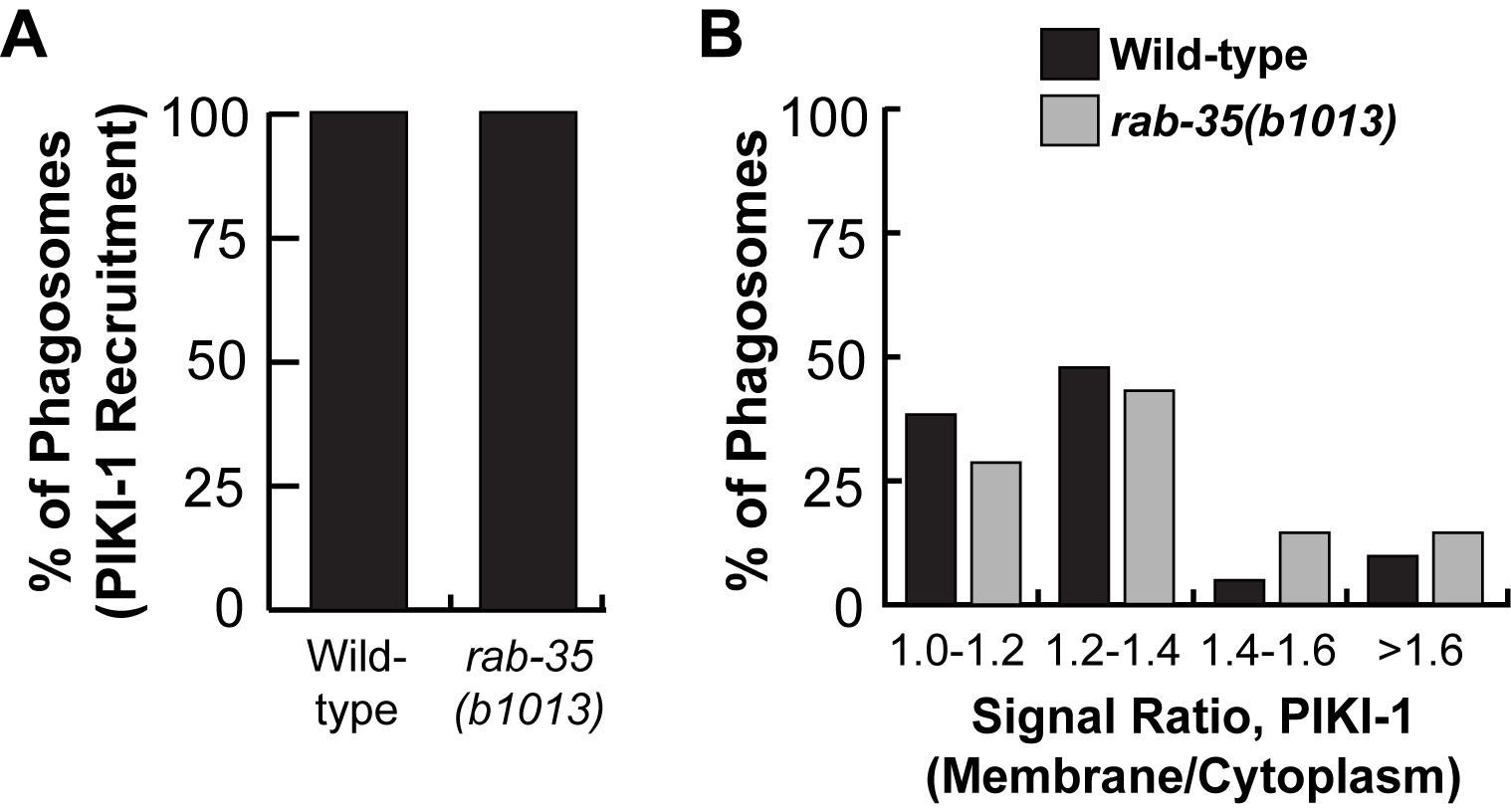

Supplement: S5 Fig — Related to Fig 7. (A) Recruitment of the GFP-tagged class II PtdIns(3)P kinase GFP::PIKI-1 to nascent phagosomes was measured using live imaging of phagosomes bearing C1, C2, and C3 in wild-type and rab-35(b1013) mutant embryos. The presence or absence of PIKI-1 on the phagosomes was scored on each phagosome and reported as a percentage for each genetic background. There was no significant decrease in the frequency of PIKI-1 recruitment in rab-35(b1013) mutants. (B) During time-lapse imaging, the intensity of the PIKI-1::GFP signal was measured on the surfaces of phagosomes containing C1, C2, or C3 and in the surrounding cytoplasm at the time point of maximal PIKI-1 phagosomal signal and the ratio of signal intensity is presented in the histogram. No statistically significant changes in the relative PIKI-1 phagosomal intensity was observed in rab-35(b1013) mutants. (TIF) [file pgen.1007558.s005.tif]

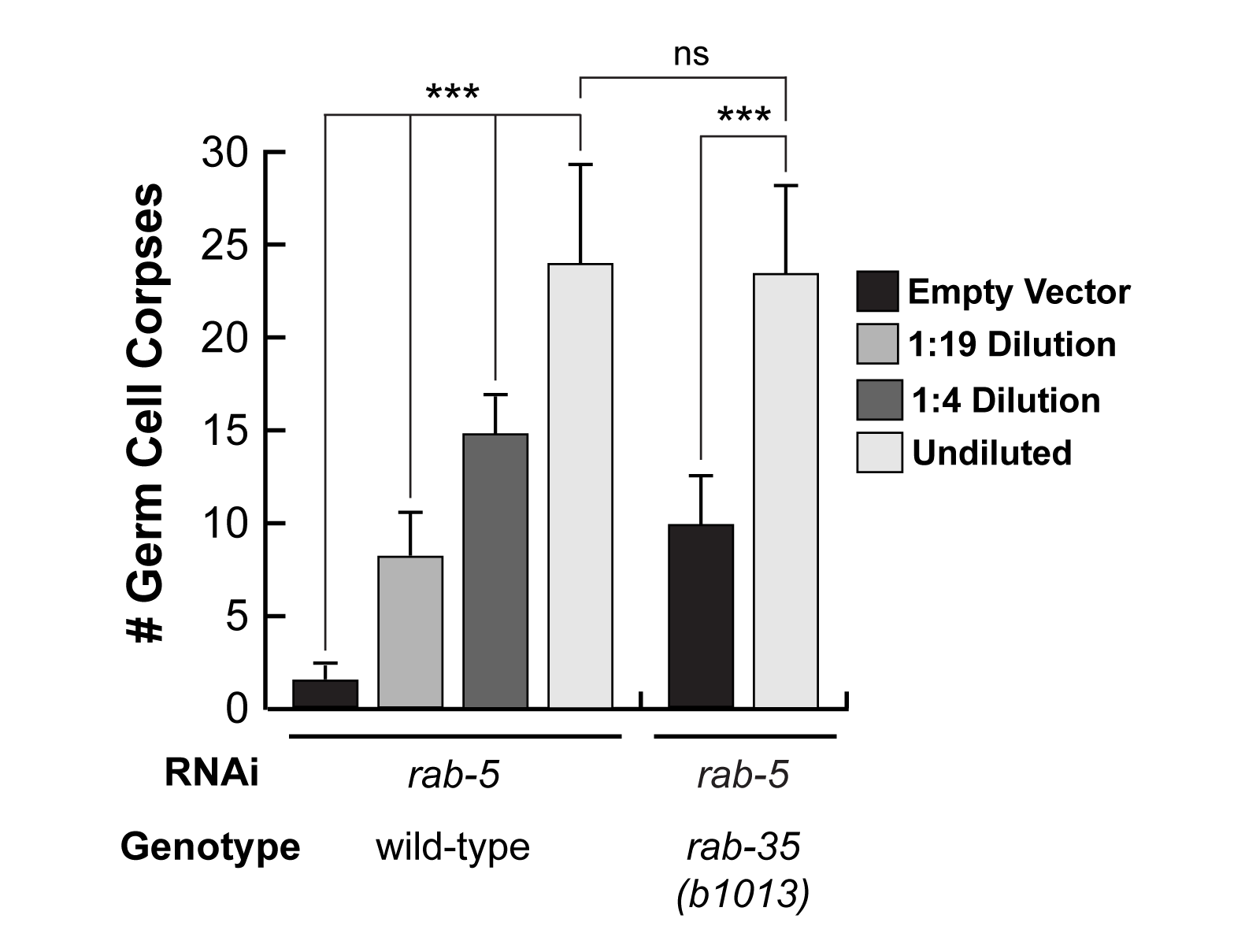

Supplement: S6 Fig — Related to Fig 8. rab-5 was inactivated by feeding wild-type or rab-35(b1013) mutant worms with different dilutions of E. coli carrying the rab-5 RNAi construct. Gonadal cell corpses were scored in one gonadal arm of each adult hermaphrodite 24 hrs-post L4 stage. Mean and sd (error bars) are presented in the bar graphs. For each sample, at least 15 animals were scored. Brackets above the bars indicate the samples that are compared by the Student t-test: *, 0.001 < p < 0.05; **, 0.00001 < p <0.001; ***, p <0.00001; ns, no significant difference. (TIF) [file pgen.1007558.s006.tif]

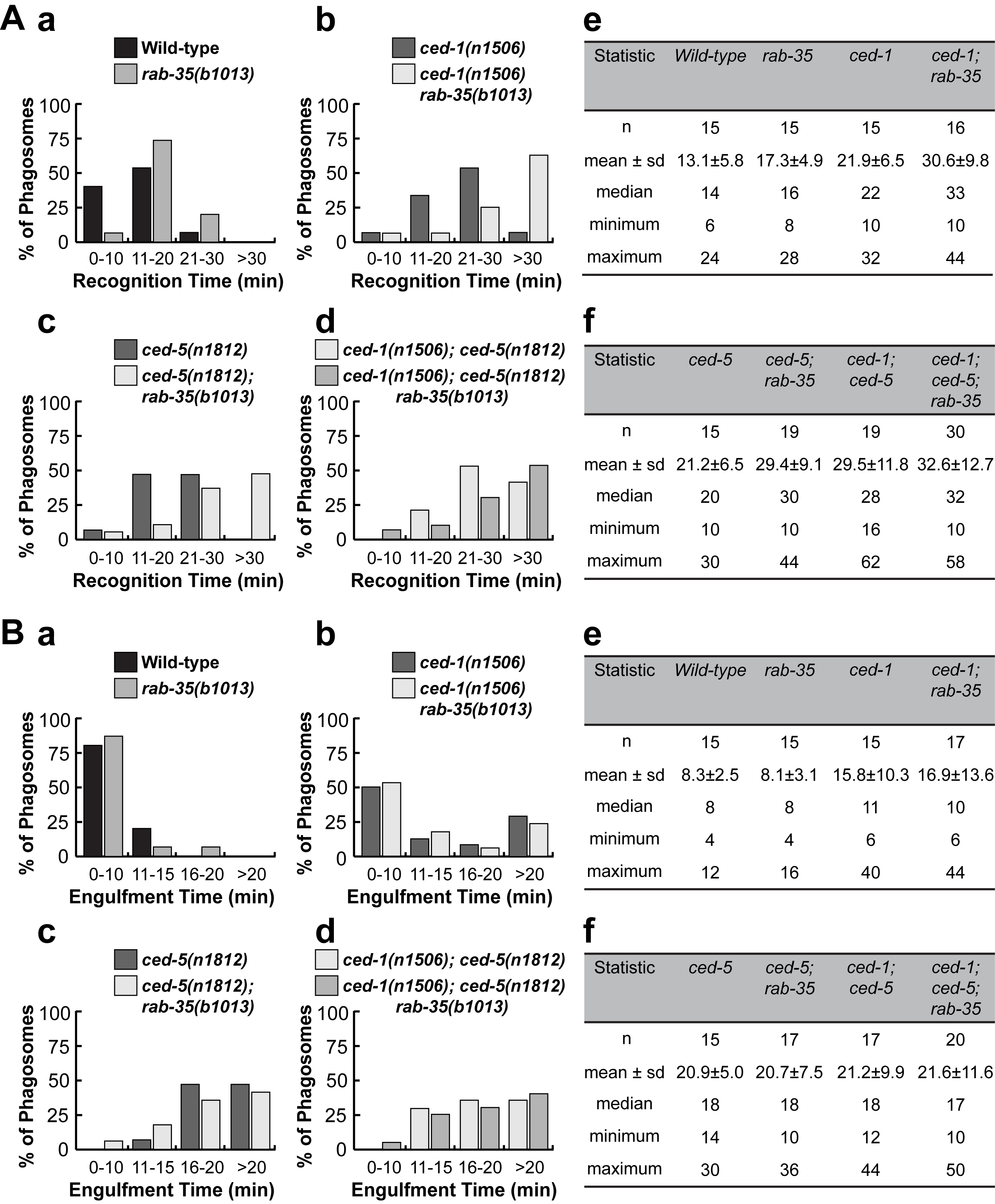

Supplement: S7 Fig — Related to Fig 9. The time it takes for engulfing cells to recognize (A) and internalize (B) cell corpses C1, C2, or C3 was determined in embryos of different genotypes using the GFP::CED-1ΔC reporter. For each strain, at least 15 engulfment events were scored. (A) The moment of recognition is defined as the first time point GFP is seen enriched in a region in contact between the engulfing and dying cell, with the moment of ventral enclosure used as a reference point (“0 min”). Histograms (a-d) and the summary (e-f) statistics are presented. (B) Internalization time is defined as the time interval between recognition of the dying cell by engulfing cells (“0 min”) and the time point that the nascent phagosome is formed. Histograms (a-d) and the summary (e-f) statistics are presented. (TIF) [file pgen.1007558.s007.tif]

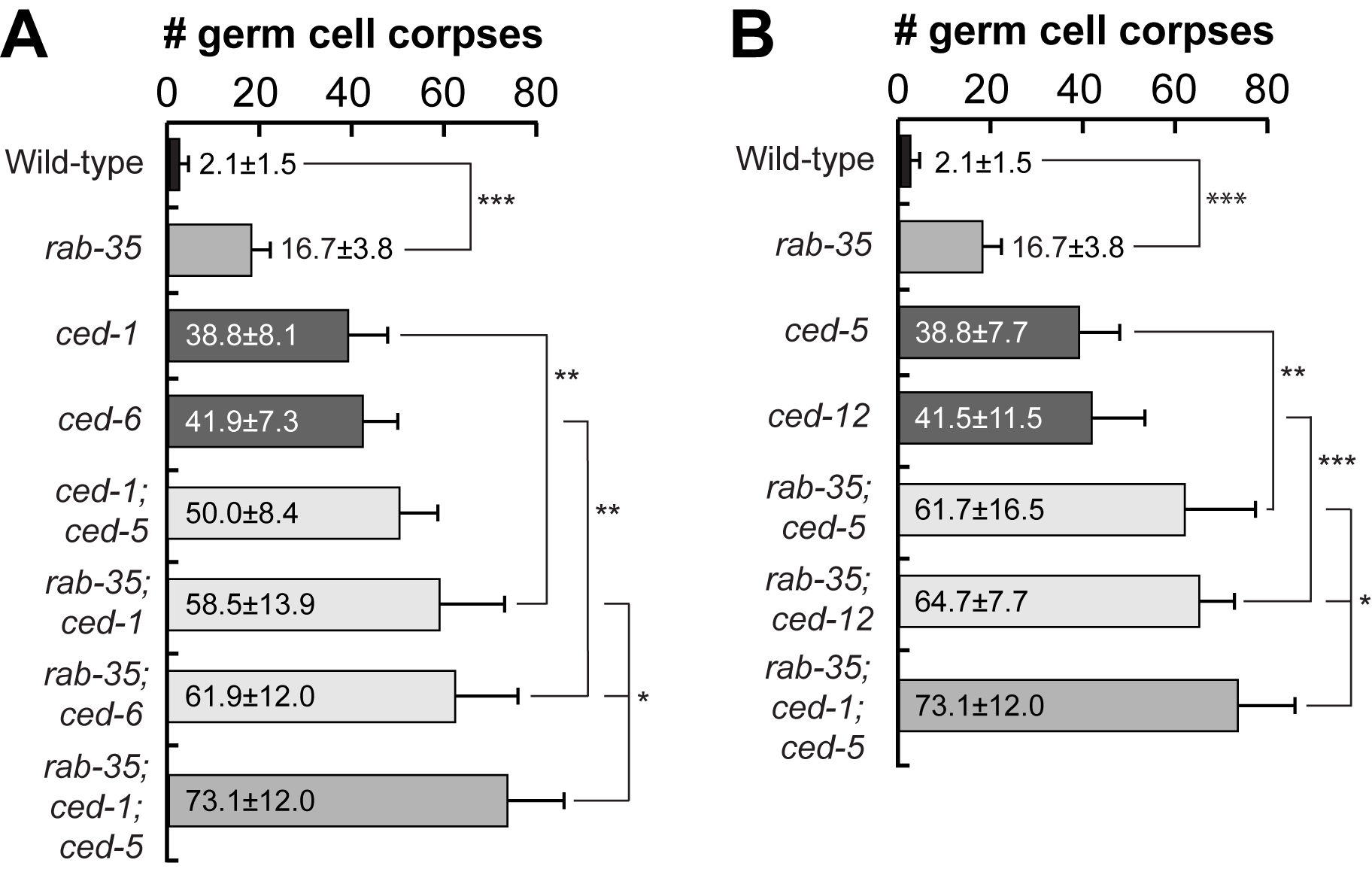

Supplement: S8 Fig — Related to Fig 10. Results of epistasis analysis performed between rab-35 and the members of the ced-1/-6/-7 (A) and the ced-2/-5/-10/-12 (B) pathways. The mean numbers of cell corpses in the 48 hour post-L4 adult gonad of various genotypes are presented in the bar graphs. Error bars indicate sd. For each strain, at least 15 animals were scored. Student t-test was used for data analysis: *, 0.001 < p < 0.05; **, 0.00001 < p <0.001; ***, p <0.00001; ns, no significant difference. (A) Null alleles [rab-35(b1013), ced-1(n1506), and ced-6(n2095)] were used. (B) Null alleles [ced-5(n1812) and ced-12(n3261)] and a severe loss-of-function allele ced-10(n1993) were used. (TIF) [file pgen.1007558.s008.tif]

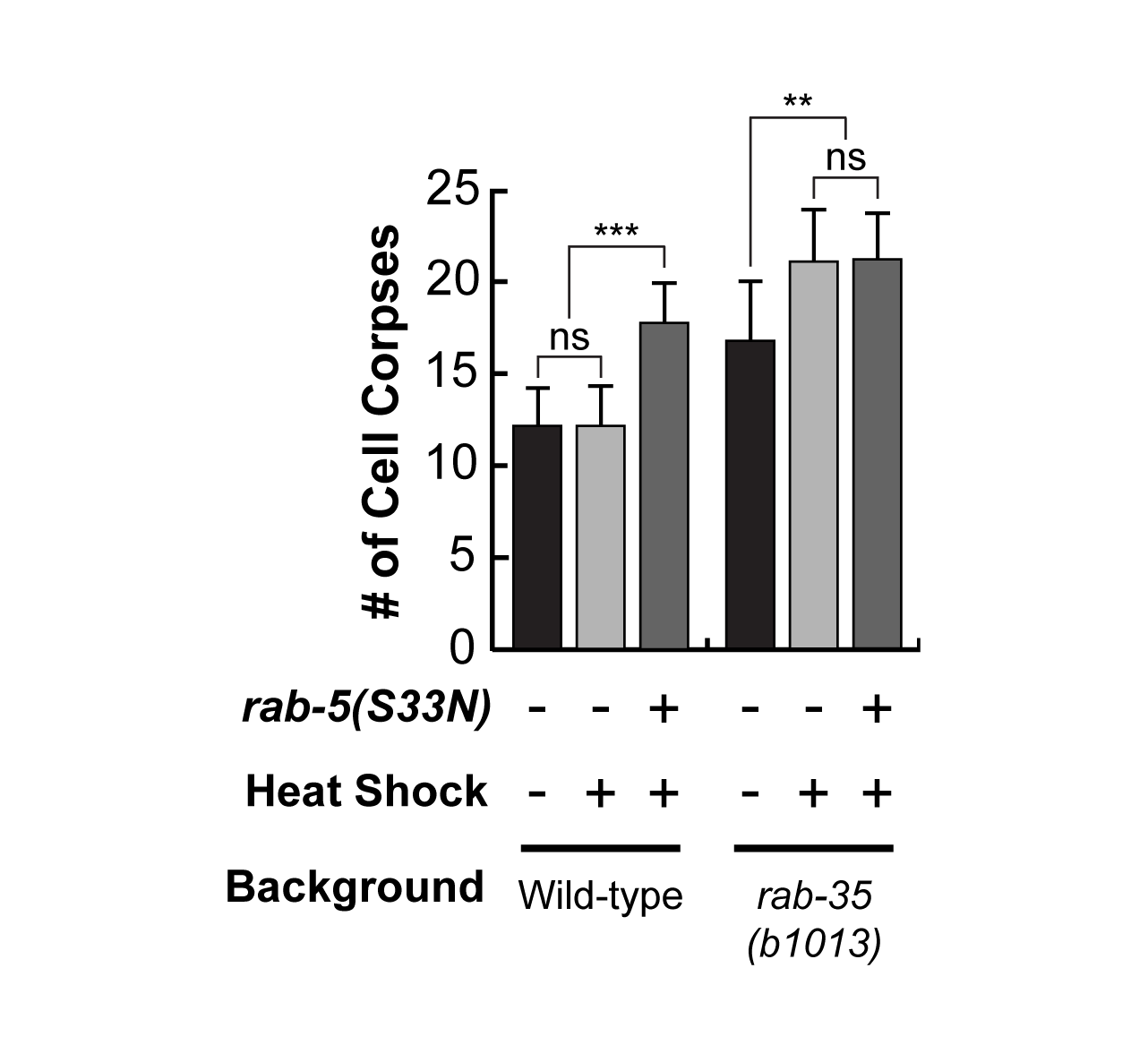

Supplement: S9 Fig — The mean numbers of apoptotic cell corpses were scored in 1.5-fold stage wild-type or rab-35(b1013) mutant embryos carrying or not carrying a transgene overexpressing dominant negative GFP::RAB-5(S33N) under a heat shock promoter, after heat shock (33°C 2 hrs) or mock treatment. For each data point, at least 15 animals were scored. Error bars indicate sd. Student t-test was used for data analysis: *, 0.001 < p < 0.05; **, 0.00001 < p <0.001; ***, p <0.00001; ns, no significant difference. (TIF) [file pgen.1007558.s009.tif]
